# Part II. Hospital Reports

**Published:** 1827-07

**Authors:** 


### PRACTICAL MEDICINE;

BEING

### The Spirit of the Medical Journals,

*Foreign and Domestic;*

WITH COMMENTARIES.

### PART II.
### HOSPITAL REPORTS.

"Ore trahit quodcunque potest, atque addit acervo."

### I. LIGATURE OF THE EXTERNAL ILIAC.
[M. Dupuytren—Hôtel Dieu.]

The celebrated surgeon above-mentioned has occupied no less than twenty-one quarto pages of letter-press (Repertoire, No. 4) with this case; a circumstance that proves he attaches no trifling importance to the operation, and to the events that succeeded. We shall endeavour to make the facts of the case stand within much narrower limits in our medical microcosm.

*Case.* F. Berger, a stone mason, aged 45 years, strained himself while lifting a heavy burthen, in June, 1815, and experienced an acute pain in his left groin for a few minutes, but which did not prevent him from following his employment at the time. Two months afterwards he felt a small tumour, two inches below the crural arch, to which he paid little attention. The tumour gradually increased in size till June, 1816, when, on making another muscular exertion, the swelling suddenly augmented to the size of a hen's egg. Finally, in the beginning of August, he fell against the edge of a large copper vessel—struck the tumour, and increased the evil. He entered the Hotel Dieu on the 23d of August, 1816. The tumour was now the size of a large pear, extending from a little above to some inches below the crural arch, standing out two inches from the surface of the parts. It pulsated in accordance with the heart. On making strong pressure on the tumour it partially disappeared, and its parietes then felt unequal, and of cartilaginous consistence. Pressure removed, the swelling returned, as it were, *per saltum*, to its original dimensions. There could be no doubt that the disease was aneurism of the femoral artery, and it was determined to try the effects of pressure before having recourse to the ligature. By means of a kind of truss, pressure was made on the extremity of the external iliac artery where it passes over the pubes, and a little above that spot. When the machine was applied, all pulsation ceased in the tangible arteries of the limb. The tumour itself was kept covered with ice in a bladder. But it was soon found that the pulsations returned in the tumour whenever the patient coughed, talked, or made even the slightest movement. Besides this, the degree of pressure necessary for suspending the force of the circulation could not be borne for any length of time, especially when the ice was applied, which rendered the pain of the pressure much more insupportable. This pressure, alternately kept up and suspended, was continued till the 18th September, when it was abandoned altogether. The tumour was now sensibly diminished. On the 20th of the same month, another bandage or belt was applied, which had the advantage of following the movements of the pelvis, and making a much more uniform and effectual pressure: but the patient had not fortitude to bear the pain for any great space, at one time, and strenuously urged M. Dupuytren to perform the operation. On the 16th October, therefore, the operation was performed in the usual way, though with considerable difficulty, in consequence of the condensation of the cellular tissue over the artery, and the enlargement of a chain of lymphatic glands. One ligature was applied an inch above the aneurismal tumour, and a spare ligature was thrown round the vessel, but not tied, still higher up on the vessel.

It was remarked during the operation, that, when the patient held in his breath, and strained the abdominal muscles, the edges of the wound were pressed together, and the peritoneum forced, as it were, through the opening. This caused considerable embarrassment.

The man supported the operation well; but, immediately afterwards, was affected with nausea and disposition to syncope, which, however, were relieved. The limb preserved its sensibility and temperature through the day; but the patient complained of pain in the abdomen, especially in the epigastric region, with constant gazeous eructations, and, at one period of the day, universal heat and some thirst. In the evening the belly was tympanitic, and the patient was in a state of extreme anxiety. There were also some symptoms of cerebral congestion in the night. *Venesection—frictions over the abdomen.* The night was spent without sleep, and in much pain. *Second day.* The motility and sensibility of the limb continued unimpaired, and the abdominal pains persisted. The stomach was so distended with gas, that it could be plainly traced across the epigastric region. The eructations were constant—pulse quick—features shrunk—tongue, teeth, and mouth, covered with black sordes. After having enemata administered, the patient had a temporary respite from suffering; but the epigastric pain returned as violent as ever. He was bled again, and more lavements were thrown up. In the evening there was some wandering of the mind, and the patient lost all recollection of what had happened through the day. Bled again to 8 ounces. Glysters brought away much flatus, but no fæcal matters. He had some hours sleep. *Third day.* The symptoms mitigated in all respects. Castor oil was given this day, but no stools procured, He slept three hours this night. *Fourth day.* Although the epigastric pain was diminished, the gazeous eructations continued. The patient had some appetite to-day. and was allowed two plates of soup. The state of the limb was satisfactory, and he slept some hours in the night, but was disturbed with frightful dreams. *Fifth day.* The dressings were removed, and suppuration was established; but there was a black spot at the upper part of the wound. The tumour was diminished to one third its original size, and had no pulsation. He had delirium in the night. Enemata brought away a copious black motion, after which the delirium ceased. *Sixth day.* The symptoms were unfavourable. The mouth and tongue black, pulse quick, something like pulsation in the tumour. The symptoms, however, got better in the evening, and the patient had some hours of sleep. *Seventh day.* The tongue dry and red—pulse not so frequent—no pain in any part of the body—suppuration abundant and good—slight pulsations, or rather tremors, in the tumour. (Legers fremissemens.) Two copious evacuations from enemata. Some appetite—some bouillie allowed. The eructation now ceased for the first time since the operation. *Ninth day.* While dressing the wound this day, a strong pulsation was perceived in the left side of the abdomen, a little above the wound, and apparently proceeding from the iliac artery. The pulsation was also more distinct in the tumour than before, although its size was daily diminishing. Some wine was now allowed to the patient. On the 13th day, a small abscess broke into the wound, and its formation was evidently the cause of the pulsation above-mentioned, and which now ceased. On the 16th day the ligatures came away. The appetite daily increased. *20th day.* The pulsations in the tumour are still visible and tangible—suppuration abundant. In the night of the 23d day, there was some hæmorrhage from the wound. When examined, the source of the bleeding could not be ascertained. *24th day.* A second and more copious hæmorrhage took place this morning, accompanied by pain in the wound. The blood seemed to come from the inferior angle of the wound, and was evidently arterial. Pressure above the wound did not arrest the hæmorrhage—pressure below did. A pad was fixed below the wound—the patient's countenance was changed for the worse—and the gazeous eructations again appeared. M. Dupuytren was puzzling himself about the source of the hæmorrhage, when he felt, on examining the abdomen, and much to his astonishment, the epigastric artery, greatly enlarged, and pulsating distinctly under the abdominal integuments. The pulsations were particularly conspicuous in the neighbourhood of the wound. It then immediately struck him that the pulsation in the tumour was owing to the free communication of the blood through the internal mammary and epigastric arteries—and that, hence, a too great facility of communication, far from being favourable to a cure, in this operation, was the cause of the reproduction of the malady.

M. Dupuytren looked upon the hæmorrhage as coming from the inferior extremity of the artery, and then asked himself the question—can this extremity be tied? It must be extremely short above the tumour, and he thought the operation impossible. If the artery were taken up below the tumour, it would also be below the origin of the profunda, and would be of no use. Should the tumour be opened, as it used to be, in the ham? How was the flow of blood to be commanded above the scene of operation, in such case? M. Dupuytren gave over all thoughts of tying the epigastric, the profunda, or the femoral artery, and determined on pressure. He, therefore, endeavoured to ascertain the exact point from which the hæmorrhage came. Pressure below the wound invariably stopped the flow of blood; while that which was made above had no effect. The blood, therefore, came from the lower extremity of the divided artery. In an hour and a half a third hæmorrhage took place, and was restrained by pressure. But pressure was painful, and whenever it was removed, the bleeding returned. The patient was in a dangerous predicament. After clearing away some clots of coagula, a rush of blood took place, and M. Dupuytren thrust his finger to the bottom of the wound, by which he arrested the hæmorrhage. He now clearly ascertained that the blood came from the lower extremity of the vessel. A dossil of charpee, well rolled in powdered rosin, was quickly introduced to the bottom of the wound, and firmly kept there by superincumbent compresses. The whole was secured by a kind of truss. This species of pressure gave great pain, but was more patiently supported than formerly, as the man was now very sensible to the danger of his own condition. In the evening, all was found secure, and no hæmorrhage had taken place. *On the 25th day*, blood was found to have escaped, and new compresses and pressure were applied. The limb was in a good condition as to temperature, sensibility, and muscular power; but the *morale* of the patient was lowered, and he felt pain in the epigastrium, and discharged much gas by the mouth. These symptoms had a good deal disappeared by the evening. *26th day.* A considerable quantity of blood was found to have escaped at the side of the compresses, as well as pus. Between the 27th and 29th days, there was some discharge of pus, but no hæmorrhage. On the 30th day, a fresh hæmorrhage occurred, but not to the amount of more than two or three ounces. It was arrested by new compresses and bandages. After this there was no more bleeding. Part of the dressings were removed on the 32d day. There was discharge of pus, unmixed with blood. On the 33d day, the upper part of the thigh was found inflamed, and the patient had fever, intense thirst, and some delirium in the night. Cold applications were used to the inflamed parts. On the 34th day, the whole of the compresses were very cautiously removed, and the wound presented only a purulent secretion, of good quality:—It was simply dressed, and the inflamed parts were kept covered with compresses, wet with the liq. plumb. acet. The aneurismal tumour had now no pulsation. There was much inflammation, however, and constitutional disturbance. Although the patient had had no stool for some days, M. Dupuytren was afraid of ordering a lavement, lest, while at stool, there might be a fresh hæmorrhage. A considerable abscess formed in the upper part of the thigh, and gave exit to a large quantity of pus. From this time the patient went on well, and by the 60th day from the operation, he was able to leave his bed.

The patient was carefully examined on the 15th January, 1827, eleven years after the operation, and was found in good health, having worked all this time at his trade, as a stone-mason.

The question of secondary hæmorrhage in this case is an interesting and important one. The ligature was applied low on the iliac, just above the origin of the epigastric and circumflexa ilii. What was the consequence? Why that, in the upper portion of the artery, there was room for the formation of a clot, two inches in length, or more; whilst, in the lower portion, the whole force of the collateral circulation was brought, by means of the enlarged epigastric, to the very verge of the ligature. Under these circumstances, no sufficient coagulum could be deposited; there was no security, in consequence, against the secondary hæmorrhage; and it was this portion of the vessel that actually and naturally gave way.—*Rev.*

### 2. DISEASE OF THE NAILS.

[Guy's Hospital.]

In the April number of the Medical and Physical Journal, we find a short communication, and a beautiful plate from Sir Astley Cooper, illustrative of the diseases of the nails, and of the structure by which they are produced. The first subject (the diseases) is contained in Sir Astley's *published* lectures, and the anatomical description has been given in his anatomical course, not published. As the communication will occupy little more than a page of our small type, we shall give it in the worthy Baronet's own words.

" *Of the Nail.*—When this part is separated by putrefaction, and its internal surface is examined, it is found to be divided into three parts: viz.—1st, a hollow and nearly smooth white surface, at its root; 2dly, a hollow white laminated surface, in its middle; 3dly, a hollow, brownish, and less distinctly laminated portion, near its extremity.

" *Of the Ungual Surface beneath the Nail.*—This is divided into two parts. Opposite to the hollow at the root of the nail is placed a highly vascular and villous surface, which I call the ungual gland, and the portion of the nail over this surface is thinner than the rest. Beyond this secreting surface appear a number of laminæ, like the under part of the mushroom, which are parallel with those placed in the inner part of the nail, and which pass in the direction of the axis of the finger. The parts of the nail usually cut project beyond these laminæ.

" The ungual gland is a very vascular surface, and its use is to secrete the nail, which proceeds from it between the laminæ placed before it; so that the nail grows from its root, as may be easily seen by cutting a notch there, which grows gradually out in about three months, advancing until it reaches the extremity of the nail. The growth of a new nail also illustrates this position.

"The laminæ situated anteriorly to the secreting surface, and upon the third phalanx of the finger, are highly vascular, as far as the adhesion of the nail extends; but beyond this the cuticle of the end of the finger turns in to unite itself to the laminæ. Their vessels are arteries and veins, the latter of which form a plexus, with very frequent communications. The nail adheres to the finger by the cuticle, and it therefore separates by putrefaction and boiling: it also adheres at its root to the secreting surface which produces it; and, above all, it adheres by its laminæ being received between the living laminæ beneath. Opposite to the root of the nail, the cutis and cuticle are double, and turn inwards; so that a considerable portion of the nail is covered by the common integuments. The cuticle unites to the nail; the cutis passes under it, to produce the secreting surface and laminæ,—it is vascular and villous, that it may secrete the nail; vascular and laminated, in order to produce the adhesion of the nail to the skin.

"*On the diseased Growth of the Nail.*—The nail sometimes grows broader than it ought, and it then produces ulceration by the pressure of its edge, which is followed by an irritable and fungous granulation. As this state arises from the breadth of the nail, and its consequent pressure, it sometimes continues for months, or even for years; yet it will yield to proper treatment in two or three weeks. The common mode of relief consists in cutting a notch in the centre of the nail; in scraping its extremity thin; in putting it frequently in warm water, and in putting a piece of lint under its projecting edge: but this mode often fails in producing a cure, and frequently is only a temporary relief. In obstinate and difficult cases of this unnatural growth of the nail, I have, for thirty-five years, recommended and practised the plan of cutting away the edge of the nail with scissors, from its extremity to its root; by which a cure is often produced in a few days, and in the worst cases in two or three weeks. A poultice only is afterwards required.

"*Of Disease in the Ungual Gland.*—In diseased states of the constitution, the secreting surface which produces the nail gets into a morbid state, and, instead of a healthy nail being formed, it throws out one which is black, everted, unadherent, and which so irritates the vascular surfaces as to produce an irritable, sloughing, and very painful sore, which renders the patient lame, so as to prevent his gaining his daily bread. As this is a constitutional as well as local disease, it becomes necessary to employ constitutional and local means of treatment. My usual plan is to give a grain of calomel, with a grain of opium, night and morning, with the decoctum sarsaparillæ compositum; and to apply the liquor calcis ℥iv. with calomel ʒj. by means of lint with oiled silk over it. This plan often succeeds; and, if it does not, it destroys the predisposition to the disease.

"After giving these constitutional remedies, if the sore does not heal, I have sometimes applied a blister to bring off the nail, and alter the action of the ulcer. But in hospital practice, where persons are anxious to return to their labour, and to have their disease quickly and effectually removed, I have always dissected away the secreting surface which produces the nail, and prevented the possibility of a recurrence of the disease." 291.

The plate which accompanies the communication is highly illustrative of the subject.

### 3. CHLORIDES OF SODA AND OF LIME.

[Lisfranc. La Pitié.]

We have already noticed M. Lisfranc's observations on the utility of this remedy in burns and in ulcers. Our readers are also acquainted with the publications and translations on this subject, contained in our last number. Some farther reports have been lately made from La Pitié, by M. E. Margot, who has been deputed to record the practice of Lisfranc, in this hospital. M. Lisfranc sets out with the following propositions.

1mo. The chlorides of soda and lime are excitant substances, capable of inducing inflammation; but it would not be prudent to apply them too near to organs, inflammations of which might be hazardous.

2ndo. These chlorides have the property, superior perhaps to all other substances, of inducing that kind of inflammation which throws out a plastic secretion quickly convertible into false membrane, and consequently producing adhesion or agglutination of the parts.

3tio. These chlorides tend to awaken the organic sensibility in portions of skin completely denuded of cellular tissue, and to cause them to adhere to the subjacent parts, when all other means have failed. Thus losses of substance are prevented in parts where, under other treatment, the cicatrization would prove a deformity.

4to. Employed for the cure of fistulæ, the chlorides, in a third degree of strength, diminish, sometimes immediately, the process of suppuration—sometimes they suppress this process instantaneously. It is in this way they prove successful. When they fail to produce this effect, their strength should be augmented—when they excite inflammation too intense, they must be discontinued for a time, and afterwards tried in a more dilute state.

5to. By these chlorides, M. Lisfranc has cured many callous fistulæ, which could not be cured by other means.

Four cases are related as examples from among a great many of a similar kind. We shall give a short abstract of these.

*Case* 1. John Sauquet, aged 27 years, was admitted into the hospital, on the 9th January, 1826. Six weeks previously he had an abscess in the loins, which was opened. A fistulous canal, more than three inches in length, existed, the skin covering which was thin, discoloured, and below the natural temperature—the sides and orifice were callous. Injections of the chloride of soda, in the third degree of strength, were prescribed, and allowed to remain for some time in the canal. These injections were renewed three times a day, and lint moistened with the solution was kept over the fistulous orifice. On the

11th the suppuration, which had been abundant, was reduced one-half. The patient had experienced some smarting in the fistula. 12th. Some degree of compression was employed. On the 14th the strength of the solution was increased—and by the 21st the cure was complete.

*Case* 2. M. Cauvait, aged 44 years, was received into La Pitié, on the 24th November, for an abscess on the upper and outer part of the leg, which had existed for two months. It had been opened, and another opening had been necessary near the outer ankle. A fistula existed between the two openings. Injections were used, but they produced no effect. Their strength was then increased, and they excited inflammation, for which poultices and fomentations were necessary. When this was over, the injections were again employed, and the cure was soon effected.

*Case* 3. A young lad was received into hospital in March 1826, for a fistula situated in the thick part of the thigh, the tract of which was four inches in length, penetrating among the muscles. This had existed for two years. Six injections of the chloride of lime were used, of the third degree, but with no effect. They were then increased in strength, and some pain was produced. In three days the suppuration was reduced one half. The injection was continued to the 26th March, when the fistula was completely cured. He was discharged a few days afterwards.

*Case* 4. Guygny, aged 52, was received on the 12th April, 1826, having been affected more than six months, with several fistulous ulcers. One was in the thigh, a little above the knee, running up four inches among the muscles. The skin covering the fistulous canal was of a blue colour. There were several other sinous sores in the same limb. She had been under treatment in the city for two or three months, without any benefit. Leeches were first applied, with poultices and fomentations; after which, the injections were used, as already described, and the patient was discharged completely cured on the 27th May.—***Revue Med. Janvier.***

Now that these chlorides have been introduced into this country, we have no doubt that they will receive attention, and be put to the test of experience.

### 4. ON BLEEDING IN THE COLD STAGE OF AGUE.*

[Royal Ordnance Hospital.]

There are few things more repugnant to the imagination of a medical man than that of venesection in the cold stage of an intermittent fever. Yet this must be from education. Books and lectures all inculcate a

* Dr. Mackintosh. Ed. Med. and Surg. Journal, April, 1827.

diametrically opposite procedure. We see the face and surface of the body pale and cold—the pulse feeble and quick—the teeth chattering—the whole body shivering—and the suffering patient huddling himself up in all the clothes he can collect, to keep the spark of life from being extinguished! The very idea of abstracting any of the vital fluid (which seems almost entirely to have vanished) is horrible. But yet, when we come to reflect that the blood has only shifted its place from the circumference to the centre, and that the internal vessels and organs must now be gorged with this fluid, and, as it were, in a state of suffocation, there is nothing very incongruous in the attempt to relieve these suffering organs, by abstracting a portion of blood from the general circulation. It was upon this idea that Dr. M. tried the effects of bleeding in the cold stage of ague, with the view of relieving the internal congestions, and moderating or preventing the subsequent reaction. But Dr. M. is not quite so original on this point as he supposes himself. Did not the Indian practitioners bleed in cholera epidemica, where the most frightful picture of the cold stage of ague is portrayed? It was Dr. Johnson who first suggested and practised this plan, in a few cases of violent sporadic cholera or *mort de chien*, as the following passage will prove.

"I have only slightly mentioned venesection, though from its instantaneous good effects in three desperate cases, I am inclined to think it might prove a powerful auxiliary, *in relieving the brain and other internal organs, when overwhelmed with blood,* EVEN ANTERIOR TO RE-ACTION; and also by moderating the violence of the re-action itself."—*Tropical Climates.*

The above was written in 1806, and published in 1811. Acting on this suggestion and experiment, the Indian Practitioners bled in cholera morbus, where the blood had so completely deserted the surface, that it was with extreme difficulty, in most cases, they could get the blood to flow in drops from a vein. This is a complete anticipation, not only of the principle but of the practice which Dr. M. now brings forward; but we freely accord to the talented writer all the credit of applying this principle and this practice to the intermittent fevers of this country, where the danger is, however, not so great, as in those terrible forms of collapse, where venesection was ventured on in the hotter regions of the earth. But to the subject before us.

Dr. M. considering justly that the difficulty of breathing, the tremors, the pain in the head, the coma, and, in short, all the remarkable and prominent features of the cold stage, are owing to congestions of blood in the brain, lungs, liver, spleen, and great vessels, determined to try the effects of bleeding, first on himself, and subsequently on others. "I have seen," says he "men in the most severe sufferings, relieved after the abstraction of six, eight, and ten ounces of blood. I have known three ounces suffice; and, on one or two occasions only I had to bleed to the extent of two pounds. The relief, which is the most perfect relief that can well be conceived, is so sudden, when a good orifice can be made, that it has surprised and delighted every one who has seen my practice."

In the year 1810, our author was himself harrassed with an ague; and bark and other remedies had entirely failed. He therefore determined to try venesection in the cold stage. Before twelve ounces of blood were abstracted, the rigors ceased, with all their unpleasant accompaniments. There was no hot stage—no sweating stage. A pleasant sense of heat succeeded the painful one of cold; and, instead of weakness, he was sensible of an acquisition of strength. Afterwards he bled many other individuals, and always with the same results. The present proofs, however, of the good effects of the practice, do not rest on his own testimony alone. The plan was put in operation in the Royal Ordnance Hospital of Edinburgh, before his pupils, and in presence of several members of the profession. Eight cases are detailed at great length; but we cannot afford space for any abstract of them in this article. We recommend a careful perusal of them in the journal where they are published. We cannot, however, deny ourselves the satisfaction of quoting the conclusions to which Dr. Mackintosh has come from the various facts and cases which he has detailed.

"1. I need scarcely say that bleeding in the cold stage will not necessarily produce death.

"2. That this practice will sometimes cure the disease; at others it will prove beneficial by breaking the chain of diseased action, and rendering the subsequent paroxysms milder and milder.

"3. That bleeding in the cold stage, in every case in which it has been yet tried, has cut short the cold fits, and has prevented the subsequent stages of the paroxysm, so that the hot and sweating stages are saved. It seems to operate by anticipating the natural efforts of the constitution, removing the internal congestion, and restoring the lost balance of the circulating system.

"4. That it promises to be most serviceable in severe autumnal intermittents; and more particularly in the pernicious and malignant fevers, as they are termed, of Italy, Holland, and other marshy countries, which are well known to be very fatal under the ordinary treatment. In these cases the reaction of the system cannot fully develop itself, in consequence of the extent to which internal congestion has taken place, and which this practice will remove.

"5. That it may be used with safety in any climate where the cold stage continues long and threatens danger.

"6. That bleeding in the cold stage is, at all events, more successful than in the hot stage, or than in the intervals. For although I have often seen bleeding used in such circumstances, and with advantage, by mitigating unpleasant symptoms, yet I have never known the subsequent paroxysm prevented by it.

"7. The practice may be adopted in the first stage of all fevers; and probably will be found useful by surgeons in concussion of the brain." 272.

We understand that some trials have been made of this procedure at the Royal Infirmary; but have not been led to believe they were quite satisfactory.

---

### 5. INFLAMMATION OF THE GALL-BLADDER, &C.
[Hôpital Cochin.]

*Case.* Madeline Bernard, aged 24 years, had been confined about three months before her entrance into the hospital above-mentioned. She was received the 20th September, 1826, and appeared to labour under merely some stomach derangement. On the 6th October, the 18th day of her illness, she presented the following phenomena:—pulse small and quick—urine yellow and turbid. *9th.* Pulse the same—skin hot —no bowel-complaint. 11*th.* The accessions of fever were quotidian —urine very high-coloured. Twelve grains of the sulphate of quinine to be taken in 24 hours. 13*th,* Diarrhœa—intermitting pulse—cough —urine yellow. 14*th,* Slight quotidian accessions of fever—decubitus dorsalis—tongue clean—abdomen painful—pulse very quick—skin very hot. 16*th,* Same symptoms—urine of an orange colour, and very turbid. 17*th,* Delirium—vomiting of every thing swallowed—no diarrhœa, no pain in the abdomen—some symptoms of bronchial inflammation, on examination by the stethoscope—pulse small, frequent, and feeble. 18*th,* Moans much—epigastric pain—urine much troubled. 21*st,* Delirium—indications of pulmonary œdema on the left side. She seemed a little better during the next two or three days. 24*th,* Extreme sensibility to pressure in the region of the liver—all the symptoms much aggravated—violent peritonitic pain in the abdomen. 27*th,* The patient threw up by vomiting a large quantity of purulent matter. Death.

*Dissection.* The gall-bladder appeared blanched externally, but on examination, presented unequivocal marks of inflammation. It was completely filled with whitish and greenish pus of good consistence—the internal surface of the organ was covered with red spots, and was evidently inflamed. Near the cervix of the gall-bladder there was a small abscess in the parietes of the organ. There were three small biliary calculi found among the purulent matter. There was no trace of inflammation in the biliary ducts, nor any affection of the liver. The mucous membrane of the stomach was softened near the cardiac extremity, but there were no traces of inflammation in this organ, or in the small intestines, till within a few feet of the cœcum, where a chain of ulcerations commenced, and reached to the colon, where there were also a few small ulcers. The left kidney was inflamed, as was also the lining membrane of the urinary bladder. The apex of the heart adhered to the pericardium by a substance evidently of the nature of a false membrane. The brain and the lungs were sound.—*Journal General de Medecine, Janvier,* 1827.

The above appears to be a case of unequivocal inflammation of the gall-bladder; but whether the fatal termination was owing to this cause, is questionable. We are disposed to consider the fever, and the intestinal ulcerations as the more immediate cause of death, though doubtless the cystitis, biliary and urinary, must have greatly added to the general constitutional fever and irritation.

6. WOUNDED ARTERIES.*

[St. Thomas's Hospital.]

Mr. Travers has recently published some cases of wounded arteries, of which we shall give a brief abstract in this article.

1. *Ligature of the Carotid.* A man was admitted on the 8th December, with a tumour on the top of the right cheek, encroaching on the orbit and even the eye-ball, attended with pain shooting towards the ear, and sense of throbbing augmented by the recumbent posture. He had cough and morning expectoration. Mr. H. Cline (1815) made an incision along the edge of the orbit, but could discover no vessel feeding the tumour. After a good deal of dissection and the removal of half the tumour, the parts were dressed. Erysipelas, fever, and delirium arose, with great pulmonary congestion and dyspnœa, &c. which were relieved by bleeding and blistering. This was on the 29th February. On the 12th April, arterial hæmorrhage took place from the remaining portion of fungus, to the amount of three pounds. It was arrested by pressure on the carotid. 13th, Mr. Travers tied the common carotid. He went on, with various vacillations, till the 24th, when strong rigors occurred, and were repeated on the 26th. On the 28th, we find the patient with delirium, subsultus tendinum—the tumour shrunk. He died the next day.

*Dissection.* The lungs were much congested, but not altered in structure. The liver and pancreas were indurated—the right kidney presented a fungous tumour on its superior surface, which involved the tubular substance. The opposite kidney was wasted. In the head, there was a considerable serous effusion between the dura mater and tunica arachnoides. The tumour was found to have no communication with the antrum or orbit. The bone was absorbed at the root of the zygoma. The tumour corresponded exactly with that found in the kidney—each being filled with a soft medullary substance.

The carotid artery was divided, and the extremities separated to a distance of three lines. Some injection thrown into the opposite carotid found its way into the upper portion, insinuating itself by the side of the clot, and filling up the space not occupied by it. The upper clot was unadherent to the internal coat, an inch and a half long, bounded by the superior thyroid artery, about half an inch short of the division. The tunics were completely severed by ulceration; but the internal tunic, separated from the middle and outer, projected above the mouth of the inferior portion of the artery, having a thin ragged appearance. The inferior clot, an inch in length, did not completely fill the tube, and was adherent only at the superior extremity to the internal tunic. There was no sign of any healthy process going forward.

2. *Subclavian Aneurism.* William Cottrell, aged 73, was admitted

* Mr. Travers. Med. and Phys. Journal, April, 1827.

on the 9th January, 1823, with a painful, pulsating tumour, the size of a Swan's egg, protruding the pectoral muscle, and extending to the clavicle. The subclavian artery was tied on the 17th January, above the clavicle. The sac having given way in the act of passing the needle, much blood was lost; and although the artery was secured, the ligature did not command the bleeding, so that it was found necessary to introduce a sponge tent into the wound, by which the hæmorrhage was controlled. On the 20th he died, the respiratory function having laboured much from the period of the operation.

*Dissection.* The lungs were sound in structure, but the right pleura was inflamed, and contained 20 ounces of serum in its duplicature. The aneurismal sac was nearly empty. No adhesion had taken place in the wound. The ligatures were found firmly seated on the artery at the root of the sac. The sac had a pouch-like enlargement upwards, which closely overlaid the artery on the pectoral side; and this having been penetrated in the passage of the needle, had occasioned the profuse arterial hæmorrhage, without saltus, and which could not be arrested by the tightening of the ligature.

Could the actual state of things have been foreseen, the operation would not, of course, have been performed. The hæmorrhage was terrific, and at one time it was expected that the patient would have died in the operation room. One would have expected that an acute inflammation of the pleura would not have been a likely occurrence, after such a depletion; yet this inflammation and its consequence, effusion, appear to have been the cause of death.

---

### 7. PAPULAR AND CRUSTACEOUS PSORIS.

[M. Alibert. Hôpital St. Louis.]

Under the above title, the celebrated Alibert designates two species of non-contagious affection of the skin, the common character of which is to induce a more or less intense pruritus, causing the individuals to scratch the parts incessantly, for the purpose of extinguishing or appeasing the terrible sensation with which they are annoyed.

### I. Psoris Papulosa.

This species presents two varieties, the P. formicans and P. pedicularis. The former has been delineated already, under the title of prurigo formicans, by M. Alibert, and is noticed in a former number of this Journal. It is with P. pedicularis we have now to do.

*P. Pedicularis.* This has been always confounded with P. formicans, observes M. Alibert, but although it has the same march and termination, it differs, by the production of an insect, which forms its essential character, and which modifies the treatment.

*Case* 1. Loyer, aged 44 years, of very sanguineous temperament, had been affected with some cutaneous complaint a few years ago; and it was for a fresh and aggravated attack that he now entered the St. Louis, covered with eruptions and devoured by vermin. There was seen issuing from beneath the epidermis, a prodigious quantity of lice. Several baths were administered; and on coming out of the water, the papulæ were found shrunk, and only brown spots remaining on the skin. When warm, and especially after the baths, he experienced the most insupportable sensations of formication. He assured M. Alibert that he felt the vermin bite and tear him far beneath the surface of the skin, particularly between the shoulders, under the armpits, on the arms, and about the knees. The patient exhaled an odour, *sui generis*, and peculiarly repugnant to the olfactories of the attendants. It was observed that the bath produced two distinct effects:—sometimes it caused the issue of a prodigious quantity of vermin, that swarmed over his body and among his clothes:—sometimes the vermin disappeared after the bath, with a great increase of the pruritus. This difference was attributed to the difference of temperature of the water.

*Case* 2. This patient was 65 years of age, and had never experienced any severe malady. About 15 months ago, there appeared on various parts of his body, a multitude of small elevations, of a red colour, and accompanied by intense itching, and an issue of pedicular vermin. The wretched man was forced to tear his skin, and yet without being able to allay the irritation, which harrassed him day and night. The sensations were most distressing when there was perspiration on the surface, which seemed to heighten the sensibility of the skin. The eruption, the irritation, and the pedicular evolution would cease for five or six weeks, and then re-appear, to continue for a month or so. The unbroken papulæ resembled those produced on the skin by intense cold—those which were torn, resembled a small scale, and were encircled with a red areola.

*Case* 3. Bernard, aged 78, had long had an eruption on his face, before he was seized, four years ago, in the middle of winter, with intense pruritus over the breast, the upper part of the back, the thighs, and scrotum. Tepid baths, blood-letting, and cooling diluents soon dispersed these symptoms; but they were renewed, with aggravation, the two following winters. In 1824, almost the whole surface of the body, except the hands and face, was covered with eruptions, and became the seat of intense itching. The papulæ, at first red, were soon excoriated, and covered with greyish crusts. When these were detached, small lice were seen to issue from the larger papulæ, and these vermin soon multiplied prodigiously. The miserable patient had scarcely an interval of repose. Each part of the body became alternately the seat of irresistible irritation, and violent stinging sensations. Towards night, especially, the unhappy Bernard was in a state of anguish insupportable, tore his flesh with his nails—and rubbed himself violently with a hard brush!

Although this poor old creature had scarcely two hours sleep each night, yet his health did not seem deteriorated, nor his appetite impaired. His digestion was good; and he had only an obstinate constipation of bowels, the usual attendant on pruritus. Venesection, leeches, opiates, warm baths, diluent drinks, and great attention to cleanliness were employed; but it required six months assiduous treatment, on this plan, to effect a complete cure. The simple warm bath produced more benefit than all the other means together. It is worthy of remark that, in proportion as the cutaneous malady gave way, the bowels became more free, and in the end, the patient had eight or ten motions daily without taking any aperient. During the last three years, Bernard has enjoyed good health, and has had only some slight itching of the skin when he takes coffee, spirituous liquors, undiluted wine, or neglects, for any length of time, the warm bath.

### II. Psoris Crustacea.

This is defined by M. Alibert, a pustular and crustaceous eruption, affecting the external parts of the thighs, arms, and sometimes the spaces between the fingers. It is often mistaken by the vulgar for the itch. It is almost always the product of want of cleanliness, or some particular avocation, and is not contagious. Its march is sometimes acute—sometimes chronic. This disease has not been sufficiently investigated, although it is very common in large cities, hospitals, manufactories, and garrisons. It generally commences in the form of large pustules, a little flattened, surrounded by reddish areolæ, which are converted into grey or yellow crusts. These pustules are sometimes vesicular, and some of them resemble the false vaccine. In general, however, they are the size of a small pea, and contain a purulent or opake fluid, resembling that of small pox.

The pustules of P. Crustacea, have a slow march, and leave a permanent mark on the skin, but not a cicatrix. The itching occasioned by these pustules is of a burning character, from the beginning, and resembles that attendant on erysipelas. The skin is tense; and the itching almost entirely ceases when the crusts become quite desiccated. This species of psoris is often developed without being productive of any disagreeable sensation. The circumstance of the pruritus going off, when the pustules have arrived at a state of maturity, is a distinction between this disease and scabies. The individuals on whom this eruption appears, are generally of a cachectic and scorbutic habit, and the cutaneous surface pale and relaxed.

Sometimes the psoris crustacea is only a temporary evil; but at other times, it is a permanent malady, or at least liable to very frequent relapses. In such cases, the skin becomes indurated and rugous. The patients generally enjoy good health in other respects, especially if the psoris is not of the acute kind. Five cases are given in illustration; but they need not be detailed in this place.

Warm baths constitute the basis of the treatment, both in the crustaceous and papular forms of the disease. The baths should be used, as detersives of the skin, and as the means of allaying irritation on the surface. Sometimes to excite gently, without irritating, emollient vapour-baths may be useful. As to internal remedies, they must vary, according to the state of the health, and the causes which have given origin to the malady. Sulphureous lotions have always, in M. Alibert's experience, tended to exasperate the cutaneous irritation. The alkaline baths, as those of Plombieres, are infinitely more useful for the papular psoris. Ointments, also, composed of the various precipitates of mercury, have proved very serviceable in M. Alibert's hands, especially for the pedicular eruption. The regimen should, in all cases, be extremely rigid. All heating, spicy, salt aliment, should be entirely proscribed.—*Bibliothèque, Janvier*, 1827.

### 8. BALSAM COPAIBA AND CUBEBS, BY ENEMA.

[Hôpital de la Faculté.]

During the last six months of 1826, M. Velpeau has tested the effects of balsam copaiba, administered by enema, in twenty or thirty cases of gonorrhœa, under the direction of M. Roux, in the hospital above-mentioned. Five of these patients were females, and all these were speedily and effectually cured. Eight cases are given at length. The results are, that the balsam, administered in the above manner, diminishes considerably the discharge in almost all cases; and, in the great majority, cures the disease completely in three, four, or more days. M. Velpeau begins with two drachms of the balsam, suspended, by means of yolk of egg, in four ounces of any bland fluid, as decoction of mallows, or gum-water; and augments the dose. The second day he doubles the quantity—the third day exhibits six drachms—the fourth eight, if the discharge continues rebellious. Some opium, in solution, is added, in order to tranquillize the rectum, and enable the patient to retain the injection, which ought to be kept in an hour or two, or till it is absorbed, if possible. Camphor was occasionally added, where the chordee was troublesome. The pipe of the syringe should be well greased, and the injection should be thrown up so as not to irritate the sphincter—that part being endued with more sensibility than higher up in the gut.

Sometimes the patient experiences no particular sensation, and retains the medicine without the least difficulty. At other times colick is produced, and the patient is unable to resist the propensity to evacuate the rectum. Sometimes the inconvenience is only temporary, and soon subsides. In very few instances was there nausea, or any general disturbance of the system. In most cases, there is produced some heat in the region of the prostate gland, and along the urethra, with sense of weight in the perineum. In one case only, some febrile symptoms were kindled up, and they appeared to be independent of the injection.

In this place, M. Velpeau relates a case which occurred under M. Bretonneau, of Tours, and which deserves notice. A young woman, after an accouchement, became affected with a large abscess in the iliac fossa, and in front of the pelvis—an abscess which opened into the bladder, the matter was discharged by the urethra in large quantities. The patient was rapidly sinking from the discharge, when M. Bretonneau exhibited, by lavements, two drachms of the balsam of copaiba in decoction of cinchona. By this practice, the quantity of matter was quickly lessened—the cavity of the abscess healed, and the patient got well.

We are now attending a man residing in Holborn, who had inflammation in the left iliac fossa, which terminated in abscess, the matter finding its way both into the bladder and rectum. The quantity is considerable, and the poor fellow is likely to sink, there being great irritability of the whole alimentary canal, and inability to take or retain food.

M. Bretonneau has employed the same mode of administering the copaiba, with success, in several cases of chronic bronchitis, accompanied by expectoration of pus, and where the patients were considered as affected with phthisis.

Finally, as we know how few individuals can bear the balsam of copaiba in the stomach, without such inconvenience as sets them completely against the remedy, we conceive that the mode here pointed out, of exhibiting this nauseous drug, will be a point gained in therapeutics. It remains to be proved, whether or not the extract of copaiba, as introduced to the profession by Mr. Thorn, will supersede the rougher form of the fluid balsam.

### CUBEBS.

This substance was tried in lavements, in doses of six or eight drachms, upon three or four patients. In one case it was completely successful, in a very rapid manner, though the gonorrhœa had continued a month. In the other cases, its effects were not satisfactorily ascertained when the paper was put to press. We have not found the cubebs disagree with the stomach so much as M. Velpeau represents it to have done with our Gallic neighbours.

---

### 9. INJURIES OF THE HEAD.

[Middlesex Hospital.]

In the April Number of the Medical and Physical Journal, a series of cases is reported from the practice of Messrs. Bell, Joberns, and Shaw, illustrating the effects and treatment of concussion, fracture, &c. of the brain and skull. We shall glance rapidly at some of these.

*Case.* 1. In this case, an Irish labourer was brought into the hospital, with all the symptoms of deep apoplexy. It was discovered that, a fortnight previously, he had been knocked down by his wife in a public house, the blow being through the medium of a half-gallon tin can. Being conveyed home, he recovered so far, in a few days, as to re-commence his usual avocations. But violent pain in the head obliged him to give up work—and in a week or ten days after this, he was conveyed to the hospital in the above-mentioned condition. On examination, there was found a wound on the forehead, under which was a portion of skull denuded and apparently dead. The process of exfoliation had commenced. The case was hopeless; but, as there were evident symptoms of oppressed brain, the only chance appeared to rest in removal of the dead bone, with the view of giving exit to any extravasation beneath. The trephine was so applied as to take in the dead bone. The patient evinced no sign of sensibility during the operation. The dura mater was slightly discoloured, and covered with sero-purulent fluid. The portion of bone removed was dead. Oiled lint was applied to the dura mater, and the integuments laid over. The man died in three hours.

On dissection, there was found a large quantity of serous fluid in the ventricles—an abscess in the right hemisphere of the cerebrum, immediately beneath the dead portion of bone—a softening of the brain in the neighbourhood of the abscess, exhibiting spots of extravasated blood.

*Case* 2. This was a boy, 13 years of age, received into hospital on the 26th June, having experienced a kick of a horse on one of his temples. There was a large wound, and, when the finger was introduced, the skull was found to be fractured, the fracture crossing the coronal suture, and thus involving the frontal and right parietal bones. There was depression of a portion of bone, a quarter of an inch below the level of the other bones. The dura mater was ruptured, as shewn by discharge of brain and blood from the wound. It was found impracticable to raise the depressed bone, without trephining; and as the boy was quite sensible—roared loudly—had a strong pulse, &c. it was determined to let the depression remain. The head was shaved, and cold lotions applied—aperient medicine was given. 27th, Passed quietly. 28th. Head-ache—dull countenance—drooping of the upper eyelids—tongue dry—skin hot. *Venesection—salts—antimony*. 29th. The antiphlogistics of yesterday produced the best effects. All the bad symptoms are gone. Discharge of brain continues. On July 2d, he was again bled. 3d. A pulsating brain-like tumour protruding from the wound, resembling an incipient fungus cerebri. 6th. The protrusion has now assumed all the characters of fungus cerebri. The pupils are dilated—skin hot and dry—pulse quick and cordy—somnolency. Simple dressing and slight pressure applied to the fungus. 8th. The patient is incoherent—pupils dilated—pulse slow and intermitting. 10th. Restless and noisy. Tore the dressings from his head. Leeches applied to the temples. He was quieter during the eleventh; but, on the 12th, the report was not favourable. He passes his urine and fæces in bed—pulse 55 and intermitting. Had small doses of calomel and antimony ter die. No increase of the fungus since the pressure was applied. 14th. The fungus is sloughing, and no disposition to fresh protrusion—is less comatose. 17th. No longer passes the urine and fæces unconsciously—large discharge of pus from the wound. From this time the boy improved rapidly; but he was detained in the hospital for several months, on account of an exfoliation of bone. The original depression continued when he left the hospital.

*Cases 3 and* 4. Two cases of compound fracture of the cranium are next related by Messrs. Bell and Shaw. The first was a boy, only five years of age, who fell into a cellar, where he remained senseless for some time. There was found a small wound of the scalp, and a fracture of the bone underneath, extending in the direction of the squamous suture, and occupying a long sweep. One portion (the upper) of the bone was depressed, and, in fact, a great part of one side of the child's skull was beaten in. The little patient laboured under the usual symptoms of concussion—as cold skin—small pulse—vomiting, &c. Cold was applied to the head. In the evening re-action ensued—and leeching and purgation were employed. *2d day:* The re-action has subsided; but the marks of violent commotion of the brain remain. The vomiting continues. On the 4th day, there was intolerantia lucis, and leeches were applied to the head—saline draughts, with antimony and opium. No unfavourable symptom occurred after this. The wound suppurated in the whole length of the fracture, and large quantities of matter were discharged. A small scale of bone was thrown off, and the child was ultimately sent out of hospital cured.

The other case was under Mr. Shaw. A female child, 4 years of age, had fallen from a considerable height, and was picked up senseless. A wound of the scalp, over the coronal suture, was the consequence, with fracture of the skull beneath, and also depression of the bone, to the extent of an inch in length. No unfavourable symptom was present. The wound was dressed with adhesive plaster, and the case went on well. Some slight inflammatory symptoms were easily controlled by calomel, antimony, and purgatives.

The reporters remark, that "the foregoing cases may be regarded as good examples of the little interference required on the part of the surgeon in elevating depressions of the skull *in children.*" We do not conceive that these cases detract from the *general rule*, which recommends the interference of art in compound fractures of the skull. It is very true that, in the above juvenile patients, all went on well. But, had bad symptoms come on, the operation could not have been performed when the parts had once inflamed. In adults, the termination would probably have been very different.

### 10. HOSPITAL REPORT FOR LAST QUARTER OF 1826.

[Hôtel Dieu.]

M. Martinet is the reporter for the wards of Professor Recamier. The mortality this quarter, in acute diseases, was about one in nine; while, of the chronic cases, one in four fell victims.

1. *Intermittent Fevers.* Several of these were extremely obstinate. and stoutly rebelled against every kind of treatment—a circumstance which has long ago induced M. Recamier to believe that many cases of ague require a certain period to run, before they can be safely stopped. In one case, there was complete want of success, and the patient left the hospital uncured. In this instance, among other means used, injections of two scruples of camphor with two grains of opium were given during the apyrexia. The ensuing paroxysm was prevented; but the succeeding attacks went on uninterrupted by any means that could be employed. In another obstinate case, the camphor lavement was followed by violent colicky pains, vomiting, and spasms. The cold stage of the succeeding attack was prevented; but not the hot and sweating stages. In some cases, after all methods of treatment had failed, the patients were left to themselves, and got well without any medicine!

2. *Intermittent Nasal Hæmorrhage.* Passing over the report on cerebral affections, which presents nothing particular, we come to a remarkable case of periodical epistaxis. The patient was a young man, 28 years of age, a ship-painter, who had been occasionally subject to nasal hæmorrhage from his youth, especially in the summer time. These epistaxes, however, were very moderate. In the month of August, 1826, the patient contracted a gonorrhœa, which was stopped by some internal medicine; after which, sores broke out around the glans penis. For these he took the hydrargyri oxymurias till the beginning of December. There was no nasal hæmorrhage from the time the gonorrhœa took place till the 18th December, when epistaxis occurred, without any evident cause, and lasted three hours, with considerable loss of blood. 19th. The bleeding returned, but it was not so great as on the preceding day. In the evening it again recurred, and he lost half a pint of blood. During the next two days, the epistaxis took place regularly mornings and evenings. He was, therefore, conducted to the Hôtel Dieu on the 22d December. On that evening he had a considerable hæmorrhage, the blood being covered with buff, and very firm. 23d. The man complains of no pain in any part of his body. His skin is pale, and inclining to yellow. Says he is extremely weak—pulse small and quick. M. Recamier, struck with the periodical character of the epistaxis, prescribed the sulphate of quinine, with some opium. But the hæmorrhage returned at 7 in the evening, and the patient lost about half a pint of blood. 24th. Return of the hæmorrhage in the morning. The nostrils were now plugged in the usual manner. No return of hæmorrhage in the evening. 25th. There was a slight disposition to bleed, which was checked by the application of some more charpee. 26th. The fetor was considerable from the plugged nostrils, and the hydrochlorate of lime, in lotion, was employed, and answered the object completely. On the 31st December, the plugs were removed. On the 4th, 7th, 10th, 11th, and 12th Jan. there were slight returns of the epistaxis, after which, the patient remained free from complaint, except debility.

3. *Hiccup, lasting* 12 *Days.* An African, named Alcinor, aged 40 years, and usually enjoying good health, had had an attack of hiccup twelve years ago, which was cured by Hoffman's liquor. On the 30th November, 1826, he was seized, without apparent cause, with a rigor, which was soon accompanied by dyspnœa and hiccup. The breathing became more and more difficult—the hiccup persisted, and was almost uninterrupted. There was no fever. On the 1st, 2d, and 3d December, the symptoms increased in violence, and on the fifth, in the evening, he was brought to the Hôtel Dieu. The sense of suffocation was now extreme. Sinapisms were applied to the feet, but produced no relief. He passed the night without sleep. 6th. He was accurately examined by M. Recamier and others, when the following symptoms were noted. Dyspnœa considerable—constant hiccup for five or six minutes, and then a short interval of repose, when the hiccup again returns. The patient cannot lie on either side, on account of the dyspnœa. He sits up constantly. Examined by the stethoscope, it was found that in the act of hiccupping, no air entered the air-cells of the lungs; but in the intervals, there was a free permeation throughout the respiratory organ. Percussion did not elicit any thing abnormal in the chest. There was some cough, with trifling expectoration. The heart's action was regular—pulse full and frequent—tongue moist and white—no tenderness of epigastrium on pressure—skin cool—countenance indicative of anxiety. Hiccup and dyspnœa continue. *Hydrocyanic acid—cupping glasses to the epigastrium.*

7th. The difficulty of breathing is increased, and the hiccup aggravated in intensity. The countenance is changed—the upper extremities cold—pulse very quick. Percussion and auscultation gave the same results as yesterday. He was bled copiously. The blood was but little buffed, but very dense. He experienced relief after the bleeding. He took ether, ammonia, and laudanum. No sleep that night. 8th. Diminution of the hiccup—respiration more free—expectoration easier—pulse nearly natural. The same medicines continued. By the 11th the hiccup and dyspnœa had entirely disappeared; but some symptoms indicative of thoracic inflammation continued for nearly a month, requiring blisters and other remedies for its removal.

4. *Articular Rheumatism.* M. Recamier thinks that the power of medicine over the course of this disease is much less than practitioners imagine. Thus, when we have driven the enemy from a joint, by means of leeches, diaphoretics, purgatives, and other medications, we have often the same enemy to combat in another part, and all our labour to go over again! That the principal seat of articular rheumatism is in the synovial membranes, there can be no doubt; but the peculiar propensity which the inflammation evinces, for shifting its seat from joint to joint, is beyond our power to explain. The reduction of the inflammation in its apparent head quarters, is no proof that we have subdued the rheumatic diathesis; and in this the disease differs materially from common inflammations of other parts. The authors of this report tried the system of depletion, local and general, in its fullest extent, without deriving any very encouraging benefits from the plan. Leeches, it is true, frequently relieve the joint or part to which they are applied, for a time, but the rheumatism returns either there, or in some other joint. Dover's powder was given in most cases. Some had perspiration, in consequence of it—others experienced no other effect than what might be expected from the opium contained in the preparation. If rheumatism, indeed, was to be cured by perspiration, there is plenty of that, in general, spontaneously produced. The reporters, however, do not mean to deny that medicine has any good effect in rheumatism. They think they have seen several cases where the cure was expedited by art. M. Recamier, being so often disappointed by other means, tried the oil of turpentine in rheumatic inflammation. Two patients out of six were relieved by the medicine; but the others experienced little or no benefit. The report terminates with the details of a case of severe sciatica, where the oil unequivocally effected a cure, after all other means had failed.

*Case.* A man, 38 years of age, had suffered during three months, with sciatica of the right thigh and leg, which had come on suddenly, and resisted every remedy which had been employed for its removal. He came into the Hôtel Dieu, on the 4th December. The pain, which was of a lancinating character, extended from the ischiatic notch to the ankle, and even to the great toe, following the course of the sciatic nerve. The pain was constant, but generally had two daily exacerbations—at noon, and in the evening. Pressure produced no mitigation of the pain, nor did it increase it. The appetite was good. Two drachms of the oil of turpentine were mixed with four ounces of honey, and the patient was ordered to take three spoonfuls of the mixture daily. Three days passed without any alteration in the complaint. The nights were spent without sleep, in consequence of the excessive pain. No effect was produced on the intestinal canal, kidneys, or other parts, by the turpentine. On the 7th, the pains being still undiminished, the medicine acted on the bowels, and produced heat in the abdomen, with some perspiration on the skin. On the 8th, the quantity of turpentine was increased to three drachms in the mixture. The patient felt heat in the stomach extending to the thighs, especially to the right thigh, with increase of urine, perspiration over the abdomen, and three stools. On the 9th and 10th the amendment was unequivocal. The patient felt great heat in the right thigh, which was covered with perspiration, though the skin felt cold to the hand of another. The left lower extremity was also covered with sweat, and the skin of that member felt warm. The urine was copious. The medicine continued. On the 11th, the patient was able to walk about the wards, almost completely free from pain. He slept soundly that night. He was considered as perfectly cured on the 12th; but the medicine was continued till the 23d December. He was discharged from the hospital on the 28th, cured.

Another patient who came into the hospital the day the above patient was discharged, and who had a double sciatica, complicated with colica pictonum, was cured in a few days by the same remedy.—*Revue Medicale, February.*

The oil of turpentine is a very disagreeable medicine to take. We have exhibited it to one patient, since we read the above report, mixed with honey, and find that it is one of the best and easiest modes of administering the turpentine. It was for melena, and the patient had a speedy recovery from the complaint.

---

### 11. HYPERTROPHIA OF THE HEART.

[M. Broussais—Val de Grace.]

In the January number of M. Broussais' Journal, there are four cases of cardiac aneurism recorded, from the wards of the VAL DE GRACE; and it is worthy of notice that, in all of these cases, there was enlargement of the liver—in three of them the enlargement was tangible below the false ribs. We do not look on these combinations as always accidental—nor do we agree with the illustrious professor that, in the cases which he records, the hepatic enlargement was *caused* by the cardiac disease. From long attention to this subject, we are induced to think that the hepatic hypertrophy is often the cause rather than the consequence of the enlargement of the heart. We shall only glance at the first case, which, we think, will tend to bear us out in the above opinion.

John Parot, aged 56 years, a sub-officer, of sedentary habits, reported that he had been ill for six months, two of which had been spent in the hospital, before M. Broussais took charge of that ward. "*The disease had commenced with pain in the epigastrium, stretching down towards the loins, augmented by walking, and incommoding the patient when attempting to go up stairs.*" He averred that he had previously enjoyed good health, except some symptoms of indigestion. On the 1st November, 1826, when M. Broussais took charge, the patient presented the following symptoms:—decubitus difficilis—pulse frequent, full, and strong—undulatory movements in the jugular veins—contractions of the heart strong, over a large space, audible to the bye-standers, without any particular noise, but more sonorous to the right than to the left. Infiltrations had commenced in the lower extremities—the eyes were yellow, as was the face, which was somewhat swelled and puffy—a tumour in the epigastric region extending to the right side, and apparently an enlargement of the liver. It was evident to M. Broussais, that the disease was beyond the power of medicine, and only temporising measures were employed. He died suddenly on the 25th of the same month.

*Dissection.* The heart was extremely dilated and hypertrophied on both sides. It was at least double its natural size. The parietes of the left ventricle were more than an inch in thickness. The liver was so enlarged as to reach below the false ribs; but its structure was not otherwise different from that of health, than being redder than natural.—There was no other disease.

From the following sentence it is evident that M. Broussais looked upon the hepatic enlargement as the *consequence* of the cardiac disease. "Le volume du foie ne devait point étonner chez un suget où la circulation se faisait *difficilement* à travers le cœur." In active aneurism of the heart, we do not see any evidence of difficulty of transmission of blood through the central organ of the circulation. On the contrary, we conceive that the circulation is much too energetically carried on through the heart;—and that this very overpowering force of the circulation is the cause of many other evils, as apoplexy, serous effusions, &c.

---

### 12. NERVES OF SENSE AND NERVES OF MOTION.

[Regimental Hospital, Dragoon Guards.]

The frequent occurrence of paralysis without loss of feeling, and of insensibility of the skin, without loss of muscular power, has long attracted notice; but it is to the experiments of Bell and Magendie that we owe the proofs of there being separate nerves for the two functions of sense and motion. Mr. Broughton, the talented surgeon of the 2d regiment of Life Guards, has lately published four or five examples of this distinction, as verified by the phenomena occurring in the living body. These were briefly as follows.

1. The first case was that of a young dragoon farrier, who came into the regimental hospital, with pain in the head and limbs, and some febrile symptoms, for which purgative medicines were prescribed. Two days afterwards, it was found that the man's speech was indistinct, and that he had some difficulty in adjusting the muscles of the face and mouth. The voluntary muscles of the limbs were in possession of their natural power; but the integuments of the left side of the body, from head to foot, had lost their sensibility, so much so, that a pin might be thrust into them without producing any sensation. The head was now leeched and blistered, and the bowels were freely purged. By these means the pain in the head and the febrile symptoms were removed, but the sensibility did not entirely return for some weeks.

2. The second patient was addicted to inebriety, and entered the hospital for an attack of pain in the head, succeeded by loss of power in the upper and lower extremities of one side, while the sensibility was perfect. There were other symptoms of cerebral affection, as giddiness, slight distortion of the facial muscles, and impediment of speech. Purgation alone removed all the symptoms, except the loss of power in the muscles, which continued, more or less, until the man was sent as a pensioner to Chelsea.

3. A trooper, 30 years of age, was siezed, while on guard, with giddiness and pain in the head, the tongue being furred and the bowels costive. He had the use of his limbs, but the integuments of the left side were insensible. The pulse being full, and up to 90, he was bled and purged freely, and then took a course of mercurial alteratives and aperients. With the improvement of his general health the sensibility of the skin returned.

4. The patient, in this instance, was seized with loss of motion in the right side of the body, with slight numbness in the face, and difficulty of adjusting the facial muscles. Sensibility was not impaired. The hepatic secretion was locked up, and the digestive organs disordered.

The warm bath and mercurial purgatives were administered, and leeches were applied to the temples. It was a good while before this man was restored to health.

These cases are curious in a physiological point of view, and corroborate the experiments made to determine the distinct origins of the nerves for sense and of those for motion.—*Med. and Phys. Journal.*

---

### 13. MERCURIAL FRICTIONS IN PUERPERAL PERITONITIS.*

[M. Velpeau—Hosp. de Perfection.]

It is quite a startling thing to see such a title as the above, at the head of a Parisian Hospital Report. Mercury in Peritonitis! This is a revolution in French practice, which we never expected to see; but so it is; and our readers will be anxious to learn the results.

M. Velpeau, after remarking that all authors are agreed respecting the fatality of the disease under consideration, proceeds to extract passages from Willis, Sauvages, Burserius, Frank, and numerous other writers on puerperal fever. His own experience confirms the melancholy reports of others:—thus in the hospital of Tours he saw eight women die of this disease—in the wards of the Hospice de Perfect. thirty—in private practice, nine—while, all this time, only *two* were saved, though the antiphlogistic treatment was energetically employed! M. Velpeau deplores the miserable feelings of the medical practitioner, when obliged to witness the inefficacy of his art against such a destructive malady. The various modes of treatment recommended by Denman, Leake, Gordon, Butter, Manning, Walsh, Hulme, Clarke, Hull, Hamilton, Armstrong, Brennan, and Sutton, were all tried—and all failed. The principles and practice of Broussais were considered to be peculiarly adapted to this disease—but the leeches failed equally with general blood-letting. It was not till after he had seen forty or fifty patients die of puerperal peritonitis, in spite of from 50 to 200 leeches on the abdomen, with all other means to boot, that our author concluded in his own mind, that the disease, once established, was rarely bettered, but often rendered worse by blood-letting, whether general or local.

Mercurial frictions, so strongly recommended by the surgeon of the public hospital of Antwerp, now only remained; but it was determined to use this remedy only as an auxiliary to blood-letting. Accordingly, in the year 1824, M. Bourgon and our author put this plan into execution, as will be seen by the following cases.

*Case* 1. A young woman, 21 years of age, was admitted into the hospital on the 29th November, 1824, having been 12 hours under labour-pains. The labour went on for upwards of 12 hours more, without any prospect of delivery, although the os uteri was widely

---

* Sur l'Emploi des Frictions Mercuriales dans le Traitement de la Peritonite des Femmes en Couches. (Clinique de l'Hospice de Perfectionnement] Par M. Alf. Velpeau.—*Revue Med. Janvier,* 1827.

dilated, and the head pretty low. The forceps were therefore applied, and the child delivered. The milk-fever took place on the 2d December, and on the 3d the patient felt herself remarkably well. On the 5th, at three o'clock, she had some altercation with her parents, and at five, was seized with a rigor, succeeded by violent pains over the abdomen. Twelve ounces of blood were taken from the arm, and 40 leeches applied to the abdomen. 6th, The abdomen is distended, and very painful throughout, especially at the epigastrium—pulse small and hard —30 leeches. At ten in the evening, an exacerbation, vomiting, flushings of the cheeks. Venesection to eight ounces—30 leeches to the abdomen. 7th, This morning, there is great prostration, delirium, constant nausea, thready pulse, abdomen greatly distended. Sanguineous evacuations could no longer be employed, and a fatal termination was indeed apparently approaching. Nevertheless, at ten o'clock at night, four drachms of mercurial ointment were rubbed over the abdomen. She died at midnight.

On dissection, there was found a considerable effusion into the peritoneal cavity—inflammation with suppuration of the uterus, fallopian tubes, ovaries, &c.

If any thing could demonstrate the inefficacy of sanguineous depletion, in this terrible disease, the above case was calculated to do so. The trial of the mercurial frictions was nugatory; for who could expect to re-animate, by any means, a patient in the agonies of death, from internal disorganization? But M. Bourgon had not courage to try the mercurial plan again, and four new victims fell before the malady, when M. Roux took charge of the Clinique. This gentleman's attention was excited by the following case.

*Case* 2. A female, who was supposed to be pregnant, was seized with the usual symptoms of acute peritonitis. Recourse was had to frictions of two drachms of mercurial ointment on the thighs, twice a day. The woman was cured in a very few days. M. Roux determined now to employ these frictions immediately after the first bleedings, in puerperal peritonitis. An opportunity soon occurred for the trial.

*Case* 3. An unmarried female, 28 years of age, of previous good health, was confined of her second child, at the hospital, on the 20th January, 1826. The labour lasted fiteen hours, and then terminated without any accident. On the 4th day, the milk febricula took place, and continued on the fifth, but without any tension or tenderness of the abdomen. During the next two days the patient went on better; but on the evening of the 7th, without any appreciable cause, she was seized with a violent rigor, and severe pains in the region of the uterus, with great fever. *Forty leeches to the hypogastrium.* 9th, The pains and fever are diminished—skin moist—diarrhœa. 10th, The pulse nearly natural, and the abdomen scarcely sensible to pressure; but, at six in the evening, the pains and the fever re-appeared. 30 *leeches to the most painful points.* 11th, The abdominal pain was diffused over a great space—the patient is extremely weak, and sanguineous emissions appear incompatible with her safety. *Fomentations.* At five in the afternoon, she considered herself better, and was placed on a sofa while the bed was made. Half an hour afterwards, she experienced a violent shiver, and at 5 o'clock the fever was very strong, the pain occupying the whole of the right side of the abdomen, and even up along the thorax to the neck, with difficulty of breathing. (Fomentations.) 12th, The whole abdomen is tense and painful. *Mercurial frictions every three hours.* After the third friction, at 9 in the evening, there was a sensible amelioration of the symptoms—the fever considerably subsided. At 11 o'clock there was a copious perspiration, and the abdomen is much softer. 13th, The pains are almost entirely gone, and the fever has disappeared. She begins to feel a coppery taste in her mouth, and the saliva begins to flow. Only one drachm of mercurial ointment is now rubbed in every four hours. In the evening, the convalescence had commenced, and next day, (14th) the frictions were discontinued. She speedily recovered.

The authors do not wish the public to attach more credit to the mercurial frictions, in this case, than they think fit; but they can say that all those, who witnessed the proceedings, were of opinion that, without this remedy, the patient would have died. At all events, the case afforded fair grounds for farther trial of the measure.

*Case* 4. Miss Le R——, aged 19 years, of delicate nervous constitution, was taken in labour, of her first child on the 22d March, 1826, having suffered considerable pain in the hypogastric region for a few days previously. Delivery took place the same evening, without accident. 23d, The patient was so well that her friends gave her nourishment, and even took her out into a chair, while her bed was made. 24th, There was fever last night, and pains occurred all over the abdomen. These symptoms, however, partly subsided in the course of the day. 25th, The milk-fever took place, and the lochia continue to flow. 26th, The breasts are swelled and painful, and the fever continues, with pains in the hypogastrium and diarrhœa. 27th, All these symptoms are aggravated—face yellowish, pulse hard, frequent, and strong—belly hot and painful, especially in the right iliac region. 30 *leeches to the abdomen.* 28th, The pain occupies the whole of the belly, which is greatly increased in size since the application of the leeches. Every liquid that is taken causes nausea and even vomiting—pulse small and frequent—face covered with clammy perspiration—alæ nasi work much—features shrunk—debility extreme—fæces pass off involuntarily—in short, every thing announced a speedy death. *Two drachms of mercurial ointment to be rubbed over the abdomen every two hours, and after the second friction, every three hours.* Mercurial action soon appeared in the mouth, and the frictions were only used six times. The symptoms were greatly mitigated that night. 29th, The diarrhœa is less troublesome—the patient slept some last night. The skin is moist, and the abdomen bears pressure without inconvenience—pulse soft, and 90 in the minute. *Three frictions prescribed,*

*with four hours interval between each.* 30th, Only two frictions were used. The patient last night threw herself into a most violent rage because she was denied wine and animal food. The countenance is now calm—pulse 80—can lie on either side—four stools—the skin is hot and dry. Pains have returned in the right iliac region. *Three frictions, of two drachms each.* These were carefully performed, and, in the evening, the pain of the abdomen had ceased, and pressure was borne without wincing. Perspiration obtains. The countenance, however, is flushed, and the pulse is feverish. The mouth being sore, the frictions are stopped. 31st, No pain in the abdomen, which is perfectly supple—pulse natural—countenance pale, but calm—appetite returned. Some broth allowed. *April* 1. The diarrhœa had ceased, and the patient is sitting up convalescent. The mouth is still very sore. 2d, Continues very well—there is some diarrhœa, from imprudence in diet, and from taking wine and oranges. 3d, The convalescence is established. Light food allowed. 4th, The patient, who was a most imperious vixen, got up and, in spite of all remonstrance, ate a hearty meal, with plenty of wine! The pulse was now small and wiry, skin hot, diarrhœa troublesome—chest painful—breathing short. Notwithstanding these symptoms, the patient could not be kept from excesses in food and wine. 5th, The abdomen was quite supple, and void of pain, on pressure. But she was delirious—respiration short and quick—pulse almost imperceptible. She died in the evening. Leave could not be obtained to examine the body.

Our authors are confident, and with reason, that the patient's death was in no way connected with the abdominal inflammation, which had evidently subsided under the mercurial influence. They are also convinced that, had it not been for the mercurial frictions, death would have taken place in less than 24 hours from the time of their first application. Their hopes were now still farther encouraged in the use of the remedy, from the trials already made.

*Case* 5. A young unmarried woman, aged 23 years, of weak constitution, but hitherto enjoying good health, was admitted into the hospital on the 30th September, 1826, in actual labour, which terminated in safe delivery ten hours afterwards. In a few hours after the birth of the child, the patient was seized with a most violent rigor. Fever followed, with slight uterine pains, and pain extending over the abdomen. *October* 1*st*. Last night was passed without sleep—belly painful, and tender on pressure, especially towards the groins—pulse full, frequent and hard—skin hot and dry—lochia almost entirely suppresed—belly very painful, independent of pressure. 40 *leeehes*. 2d. The bites bled all night—belly less sensible on pressure, but is much more tumefied, and the pain more generally diffused—pulse feeble, but less frequent—lochia have not re-appeared. The debility is now too great, and the pain too much diffused, to warrant the re-application of leeches. At mid-day, a violent rigor, acute pains in the abdomen, nausea, pale countenance, small pulse—great tendency to syncope, from the loss of blood by the leech-bites. *Two drachms of mercurial ointment to be rubbed over the abdomen every three hours.* There was great difficulty in effecting the first friction, in consequence of the tenderness of the abdomen. The second friction was better supported, and, even then, the symptoms appeared somewhat mitigated. The countenance, however, continued sunk, and the nausea distressing. The skin began to relax a little. Before the third friction was employed, a coppery taste was felt in the mouth, which this third friction augmented, even to slight ptyalism. 3d. The patient is better in all respects. Mouth very sore, and gums greatly swelled. To stop the frictions. From this time she rapidly recovered, and convalescence was complete on the 6th. She was discharged the hospital on the 17th October.

*Case* 6. Pichard, aged 21 years, of sound constitution, was admitted into the hospital on the 18th of October, 1826, having been some hours in labour. She was not delivered till five o'clock in the morning of the 23d, but then naturally and without any accident. 24th. Had sharp pains in the belly last night—fever to-day, with hot skin and full pulse. Five o'clock in the evening, the abdominal pain very acute—fever very strong. *Venesection to* 12 *ounces.* Nine in the evening, no amelioration. 40 *leeches to the abdomen.* 25th. The pain extends over the whole abdomen—constant nausea—restlessness—extreme agitation—excessive debility. 30 *leeches.* Six o'clock in the evening; vomiting—great agitation—abdomen tumid—countenance exsanguious—pulse very small—the leech-bites still bleeding. Between this and ten o'clock she had shiverings—excessive abdominal pain—belly greatly distended. *Two drachms of mercurial ointment in friction every three hours.* After the first friction the pain was mitigated, but the nausea and retchings continued. After the third friction the patient had some sleep. 26th. Lies on one side semibent—abdomen softer, and painful only on pressure. *Another mercurial friction.* Mouth not affected. In the evening, the pain returned in the iliac regions—pulse small—violent shiverings, like the first stage of an ague—at eight o'clock, perspiration—inability to lie on the side—abdomen painful throughout. *Another mercurial friction.* 27th. A shivering fit at seven this morning—features shrunk——some nausea—abdomen rather less sensible—at eight in the evening, some moisture on the skin. *Warm bath.* At midnight, had the rigors again—at five in the morning, a violent shivering—abdominal pains greatly augmented—slight delirium—at six o'clock perspiration. At seven in the morning, screamings—great restlessness. *Three frictions had been used in the course of the night.* 28th, A little more calm this morning—pulse better—abdomen softer, but still painful. *Mercurial friction—two grains of calomel every two hours.* She continued better through the day; but in the evening the shiverings returned, with some nausea and slight delirium—abdomen very tender. 29th, Decubitus lateralis—abdomen soft, not tender even on strong pressure—tongue cleaning—mouth beginning to be sore:—warm bath—to stop the mercurials. In the evening, a rigor stronger than any which had preceded—diarrhœa—some sleep in the night. 30th, The abdomen is again tense and painful. At mid-day, all the symptoms assumed a dangerous aspect, and at midnight she sunk.

*Dissection.*—In the head and chest every thing was sound. The mucous membrane of the stomach and bowels was pale throughout. There was an effusion of yellowish serum to the amount of about two pints in the abdomen—the uterus was in a state of suppuration, both on the inside, and in the parietes of the organ. The ovarian veins were full of pus, forming two cords as large each as the finger, "extending from the sides of the uterus to the emulgent veins."

*Case* 7. Mary D——, aged 25 years, strong and of large stature, was taken in labour of her second child, on the 18th November, at four in the afternoon. She was received into the hospital on the same night, and safely delivered the next evening. 20th, The patient is very comfortable; but, at six in the evening, she was seized with a rigor, acute pain in the hypogastrium, and soon after throughout the whole abdomen. *Bled to* 15 *ounces.* The pain augmenting, *sixty leeches were applied at midnight over the abdomen.* At two in the morning, the abdomen was distended—frequent nausea—general pain over the abdomen. 21st, The leech-bites are still bleeding—abdominal meteorism still considerable—pains very acute—face pale—features shrunk—breathing short—pulse small, irregular—constant tendency to syncope—lochia suppressed—the least motion or pressure caused violent pain in the abdomen. It seemed unlikely that she would live through the day. *Mercurial frictions every two or three hours.* At five in the evening, the abdominal pains are less acute—pulse and countenance improved. 22nd, The abdomen, though much distended, is softer, and less tender on pressure—the nausea continues—cheeks of a purple colour. *The frictions continued.* In the evening, vomiting of greenish matters, but, in all other respects, she is better. 23rd, The nausea has ceased—the abdomen is still softer and less painful than yesterday—diarrhœa. In the night she complained of violent pain in the left lower extremity. 24th, Considerably better—no pain whatever in the abdomen, even on pressure—the belly much diminished in size—pulse nearly natural—mouth not affected by the mercury. 25th, Two mercurial frictions have been made during the last 24 hours—symptoms of peritonitis have re-appeared—frictions continued—in the night an abundant perspiration. 26th, Much better—the left lower extremity still painful and swelled. This day the mercurial frictions were irregularly performed—in the evening, acute pain in the right groin—an abscess forming in the calf of the left leg—15 leeches to the part inflamed—the frictions are made on the thighs, as the skin of the abdomen is tender.—27th, The night was calm, but without sleep—the abdomen is rather hard and distended—no diarrhœa—pulse small and irregular—features contracted. She sunk at four o'clock on the morning of the 28th.

*Dissection.* Slight traces of inflammation in the chest—about a pint of serous effusion, with albuminous flocculi, in the abdomen—the uterus was about the size of a child's head, its internal surface being lined by a layer of albuminous and putrid-looking matter, of a black colour—parietes of the uterus contained numerous purulent depôts, and many of the uterine veins were filled with pus. All other internal parts were sound.

Such are the facts which our authors have laid before their brethren —concealing none of the failures, while portraying what they conceive to be evidence in favour of the treatment now recommended for trial in this fatal malady.

The remarks which they have appended to this paper, relate chiefly to the sixth and seventh cases. They fairly own that they believe the principal cause of want of success, was owing to their not pushing the mercurial frictions far enough, or keeping them up with vigour adequate to the urgency of the danger. They are quite satisfied, and think it must be evident to all, that the sanguineous depletion totally failed in these cases, if it did not occasion actual harm; while the mercurial frictions always mitigated the abdominal pain and tension, and, on more than one occasion, seemed to place the patients out of actual danger. They are free to confess that it is probable the utility of the frictions may be increased by combining with it the internal use of calomel and opium, of which, however, they have not yet had experience.

They think themselves authorised to draw the following conclusions from the facts which they have already observed.

1mo, That puerperal peritonitis, once completely established, and abandoned to itself, is almost invariably mortal.

2ndo, That it remains yet to be proved that sanguineous depletion *alone* is adequate to the cure of the disease.

3tio, That the writings of Hamilton, Gordon, and Vandenzande incontestibly prove that, by means of calomel, in large doses, many cases of puerperal peritonitis, of the most severe kind, have been saved.

4to, That mercurial frictions over the abdomen, made at short intervals, promise considerable success, and that this ought to draw the attention of the profession to the said point of practice.

5to. That, by means of mercurial frictions, patients have been rescued, as it were, from the brink of the grave; and consequently that this remedy should be tried, however, late in the disease.

6to, That the friction should be continued, without fear, until the mouth becomes affected—and, in most cases, for some time after all the symptoms have subsided.

7timo, That it would probably be advantageous to conjoin the internal administration of calomel, warm baths, and warm temperature.

8vo. That the facts observed by the authors, without being sufficiently numerous or conclusive, are yet such as may encourage, authorise, nay compel practitioners to prosecute the enquiry.

In conclusion, we have only to remark that the cases detailed above, with the post-mortem researches, together with others which have fallen under our own notice, convince us that, in the majority of cases, the uterus is the primary organ inflamed, and that the sanguineous depletion should be made as near as possible to this focus of the disease, especially at the very beginning. The groins, the hypogastric region, the labia pudendi—nay, the vagina itself should be freely leeched—and why should not cold be applied to the region of the uterus, when we see how efficacious it is in inflammations of the brain? Mercurial frictions ought to be commenced immediately after the first local bleeding, and continued till ptyalism take place, or till the symptoms are completely subdued. We agree with our authors that, when the peritoneal surface is involved in the inflammation, frictions over the abdomen are far more likely to succeed, than frictions on the thighs.

---

### 14. ON THE OPERATION FOR CATARACT.

[M. Royer Collard—Hôtel Dieu.]

Three modes of giving the rays of light a free passage to the retina, when obstructed by opacity of the cristalline lens or its capsule, have been adopted—1st, By extraction through an opening in the transparent cornea—2nd, By depression, or slicing, with a needle introduced through the sclerotic—and 3dly, by KERATONYXIS, which is depression or cutting up of the cataract by a needle pushed through the transparent cornea. It is on this last operation that the present hospital report from the Hôtel Dieu is founded.

#### KERATONYXIS.

It is 23 years since Dupuytren performed this operation, by accident, on a young girl, whose eye he could not fix for the common operation of couching, and therefore he transfixed the transparent cornea with the needle—pushed it through the pupil—broke the capsule of the lens—and cured his patient. He was not then aware that this operation had been performed in other countries; but when he found that Keratonyxis was so much esteemed in Germany, he put it to the sure test of experience in the HÔTEL DIEU, and charged M. R. Collard with the task of recording the results.

After a great number of trials, M. Dupuytren came to the following conclusions, viz. that the operation in question (Keratonyxis) is not more easy of execution than that in which the sclerotica is punctured—that it is a trifling advantage to be able to perform this operation on both eyes with the same hand—that, in this process, the position of the operator's hand between his own eye and that of the patient, is a disadvantage—that the narrow circle of the pupil limits the movements of the needle, and prevents the operator from readily displacing the cataract, and separating the adhesions of the lens with the ciliary processes—that this mode of operating does not prevent either the nervous or inflammatory accidents which sometimes follow couching—that it exposes to iritis as much as, perhaps more than puncture of the sclerotica—that it is sometimes followed by opacity of the cornea, at the spot where this membrane is punctured—and finally that, *cæteris paribus,* this operation does not differ, in any sensible degree, as to its results, from the other mode of operating through the sclerotica, *in the majority of cases;* but that, in particular cases, it has its advantages, and therefore should not be abandoned.

M. Dupuytren attaches great importance to preparatory measures in this operation, which consist of venesection, purgation, leeches, blisters, antispasmodics, &c. according to the constitution of the patient. Some drops of the solution of belladonna or of laurel water are introduced between the eye-lids the evening before the operation, and the unaffected eye is covered with a bandage during the operation.

The patient being properly placed in bed, and the eye-lids separated by his assistant and himself, he plunges the point of the needle through the cornea on a level with the lower edge of the dilated pupil, and having advanced the instrument through this aperture, he cuts up, or displaces the crystalline, en masse, as he judges expedient. The operation done, he covers both eyes with a bandage, and carefully excludes all light from the patient's room, prescribing the lowest diet, and absolute repose. The symptoms which succeed are narrowly watched and promptly met by the proper antiphlogistic means. The following are the results of 21 operations by the Keratonyxis:—Eleven were speedily and durably successful—six required a month for the cure—two cases were followed by nervous accidents—five of the patients had slight ophthalmia—two had iritis—four were operated on twice or thrice—one lost the eye entirely by inflammation—and one lost sight by the formation of a cicatrix on the cornea, in front of the pupil.

In fine, the cases where this mode of operating is advantageous, according to M. Dupuytren, are very few; such as where the edges of the orbit are very prominent—where the eye-lids cannot be opened sufficiently wide—where the eye is very small, and deeply sunk in the orbit—where there is excessive mobility of the eye-ball, and especially where there are muscular movements, particularly in young people, which would be likely to embarrass the operator.—*Repertoire*, No. 4.

---

### 15. CAROTID ANEURISM—LIGATURE BEYOND THE TUMOUR.

Our readers will remember that in our Number for April, 1826, we gave a very full account of the operation performed by Mr. Wardrop, for carotid aneurism, to wit, the application of a ligature on the artery beyond the tumour. We thought then, and we think still, that the practice in question was "honourable to surgery, and creditable to the surgeon." Others, however, held a different opinion; and, as circumstances have turned up since, which would seem to throw some degree of discredit on the results of the operation and conclusions of the operator, we think that we shall do right in giving them a full and impartial consideration.

In No. 173 of the Lancet is detailed a second case of Mr. Wardrop's, in which the same plan of treatment was pursued.

*Dec.* 2. E. B. æt. 57, always enjoyed good health till four years ago, when, after severe pains in the head, she experienced a fit of apoplexy, but recovered under the use of bleedings, blisters, &c. Two years afterwards, she had a similar attack, which was relieved by similar treatment. About six months since, a pulsation was accidentally discovered in the neck, which a surgeon pronounced to be aneurism of the right carotid artery, and proposed an operation; but the patient would not submit to it.

On the right side of the neck, immediately beneath the sterno-cleido, the two portions of which are parted over it, is a strongly pulsating tumour, about two inches and a half in breadth. The pulsation extends from the clavicle, about two inches upwards, and is much stronger above than below. The patient has had a hernia in the right groin for four years, and both legs are œdematous, tense, and painful. She has severe head-ache, and is unwilling to lie on the right side, as the pulsation in the tumour is thereby increased, together with much noise in the corresponding ear—sleep disturbed—appetite bad—thirst great—pulse has a slight thrill—bowels confined. A pill of hydr. submur., antimony and extractum rhei, was given at bed-time. On the 3d, she lost ℥vj. of blood from the arm. 4th. Urine scanty and high-coloured. *Pil. scillæ*, ʒj., *Pil. hydr.* gr. xij., *Pulv. digitalis*, gr. iv. ℞ *ft. pil.* xij. *capt.* j. *ter die.* 6th. Urine more copious—legs smaller—rest disturbed. Two grains of opium at night. 7th. Tongue brown—thirst—sickness. *To take* j. gr. *of opium.* 8th. Better—tongue still brown. *Salts and senna.* 9th. Bowels regular—felt very giddy for some minutes this morning, and nearly fell from her seat.

On the 10th, "two grains of opium having been given in the morning, Mr. Wardrop, with the assistance of Mr. Lawrence, and in the presence of many practitioners, tied the carotid artery at its emergence from beneath the omo-hyoideus, and above the tumour. As the patient's neck was fat, the incision in the integuments was not less than three inches in length; the rest of the operation was chiefly accomplished by means of a silver knife, and not above a table-spoonful of blood was lost. The ligature, formed of a portion of silk-worm gut, as recommended by Mr. Fielding, was readily conveyed round the artery by Bremner's needle, and, after being tied, both ends were cut away. The external wound was secured by two stitches and a strap of adhesive plaster." P. 396. The patient felt only a slight faintness after the operation—the pulse of the *right* wrist continued full and strong, that of the *left* was feeble. *Vesp.* A little dryness in the fauces, which is attributed to the opium. *Calomel*, gr. v. 11th. Slept well—"pulsation in the tumour much reduced, particularly in the tracheal portion; pulsation of the opposite carotid increased in force." On taking away the adhesive plaster, the wound was found to adhere. 12th. Pulse of the right arm still stronger than that of the left—pulsation of the left carotid increased—the right temporal artery beats feebly. The tumour is as yesterday—it is easily diminishable by gentle pressure, but this causes head-ache. Had faintness twice yesterday afternoon, with pain in the bowels, which are confined. *Salts and senna immediately.* 13th. Better. She has not now the sense of suffocation on lying down on the "left" side, which she used to have.

Pulsation in the external part of the tumour, on pressing which, the pains in the head are brought on as violently as ever. *Pil. colocynth. c.* ℈j., *Calomel*, gr. iij., *M. ft. pil.* v., *capt.* j. *mane et vespere.* Dec. 21. No pain in the head—appetite—feels "quite comfortable." So ended the report, and practitioners were invited to repair, as soon as might be, to Panton Square, as the woman was expected soon to leave the hospital.

Now, upon this case, we shall take the liberty of offering a few remarks, made, we beg to be understood, with a feeling of the utmost good-will to Mr. Wardrop, and respect for his abilities. We think then, that Mr. Wardrop was not doing justice to himself in selecting the above case for operation. A patient evidently out of health, affected with hernia, and "both legs œdematous, tense, and painful," is no subject for any great operation, much less for a new and almost untried one. The mere fact of there being a pulsating tumour in the neck, is not a sufficient warranty for its being an aneurism of the carotid, and of that only; and we are confident it cannot be denied that the sense of suffocation on assuming the recumbent posture—the head-aches—the ill-health, and, most of all, the œdema of the extremities, indicated (what was afterwards too fully proved by the dissection) disease of the heart. The stethoscope might have settled the point, but that was not applied. The Lancet, however, published on the 23d, (thirteen days after the operation) took a more comprehensive view of the subject, overlooked all the above circumstances, and gazetted the case as quite successful.* Thus matters stood till April, 1827, when, in the number of the Medical and Physical Journal for that month, there appeared the following article.

"*Dissection of one of the Cases of Aneurism in which the Carotid Artery was supposed to have been tied beyond the Tumor.*

"Our readers are probably aware that it was proposed by Dessault to tie the artery, in certain cases of aneurism, beyond the tumor, and that this operation was actually performed by Deschamps and by Sir A. Cooper; but, proving unsuccessful with them, never became generally adopted. Allusion is made in the present Number of the Journal to Mr. Wardrop's attempt to revive this method of operating; and we therefore think it right to make our readers acquainted with the state of parts, as discovered on the post-mortem examination of one of the recent cases.

"The patient alluded to died last week, and the body was examined on the 23d, when it was found that *the carotid artery was pervious and*

---

* This is not an uncommon failing with the Lancet. Some time ago it reported a case from Panton Square, where, as it stated, an old ununited fracture of the humerus had been quite cured by Mr. Wardrop, by means of the seton, as practised by Dr. Physic, of Philadelphia. The man entered St. George's Hospital, almost immediately afterwards, with the arm nearly as bad as ever it was. He is now *really* under cure by means of pressure, employed under the direction of Mr. Brodie.

P 2

*undisturbed, presenting one continuous tube throughout, there being no unusual appearance, and no aneurism.* The heart was affected with Hypertrophy.

"Mr. Travers, with reference to the alledged success of this method, remarks, (page 331,) that it will be of much importance '*if borne out by similar results;*' and we have given the above details because it is obviously of great importance that surgeons should be able to form a true estimate of the value of any proposed method of treatment as soon as possible, that it may either be rejected or adopted, according to circumstances.

"We are quite aware that mistakes will sometimes happen, even in the hands of skilful surgeons; and it is this consideration which has induced us to withhold *numerous other instances of unfortunate operations,* which have been transmitted to us for the purpose of publication, because they have not, like the present case, been connected with any important practical question." 376.

In the Lancet, also, of the succeeding week, No. 188, the subject was taken up. It is there stated that, three weeks after the operation, the patient caught cold and drank some spirits, after which there came on cough and fever; the tumour increased in size and the pulsation became stronger. By the aid of small bleedings, she appeared to become convalescent, but in three weeks more, "together with all the symptoms of hypertrophy of the heart, indicated by a stethoscopic examination," there took place much œdema of the legs, which continued to augment in spite of diuretics, &c. and, on the 23d March, the woman died. All this time she had not complained of her neck, nor of any symptoms of disturbed circulation within the head. "Up to the day of her death, a tumour remained in her neck of about the bulk of an almond, which pulsated strongly, felt very thin in its coats, and its contents could be readily squeezed out of it, but returned rapidly when the pressure was removed.

"*Morbid Anatomy.* This interesting dissection was conducted by Mr. Bennett, of which he drew up the following memorandum:—

"'The body was generally anasarcous, the lower extremities being swollen to twice their natural size. The tumour, which had existed in the neck during life, was not apparent after death. The arterial system being that to which our attention was particularly directed, the thorax was opened, and the teguments of the neck removed. The heart presented an unusual state of *hypertrophia* of both sides, being three times as large as natural. The serous membrane contained the usual quantity of serum found in similar cases, and appeared to have suffered from repeated attacks of inflammation, judging from the extent and thickness of the patches of organised lymph which had been previously effused from time to time. The aorta and arteria innominata presented nothing remarkable externally, but, internally, innumerable small yellow patches were observed, where ossific depositions had commenced. The muscles of the neck being removed, the arteria innominata, right subclavian, and carotid, were exposed. The carotid artery, immediately after its commencement, presented a manifest dilatation, which corresponded with the situation of the tumour that existed prior to death; and the fact of the vessels being perfectly empty, and their walls collapsed, readily accounted for the swelling having disappeared. The length of the dilated portion, was a little more than an inch. On examining the upper part of the artery, and replacing the teguments, so as to compare the relative positions of the cicatrix, (where the wound of the operation had been made,) and that of the vessel, both were found to be in exact correspondence; the line of the cicatrix obliquely crossing nearly at its centre, the line of the artery about half an inch below its bifurcation into the external and internal carotids. Intermediately between the cicatrix and the artery, the space was occupied by cellular tissue, condensed into a mass of apparently a fibrous structure. This substance intimately adhered not only to the artery, but also to the eighth pair of nerves. On slitting open the artery, the dilated portion was much thinner in its wall than the rest of the vessel. The internal surface presented numerous patches, or rather elevated flakes, of a yellow substance, but no cicatrix, or other appearance, could enable us to ascertain the precise point where the ligature had been applied. The carotid was completely pervious, as also were its several branches, with the exception of the superior thyroid, which was filled with a plug of organised lymph. It was also found that the vertebral artery, on the same side, was filled with a similar plug.'" 29.

Between these two statements there are some obvious contradictions; but on which side (for both cannot be strictly correct) the inaccuracies lie, it is for others to say. To avoid, however, all appearance of unfairness, we shall ground what observations we have to make on the report as *officially* promulgated in the Lancet.

The first question which presents itself is—the propriety or impropriety of performing the operation at all. On this subject, there can *now* be but one opinion. The dissection has proved most unequivocally that there existed, what was in fact pretty evident from the symptoms during life, hypertrophy of the heart, at the time the operation took place. But this dissection has done more, for it has proved that there was no aneurism of the carotid artery. It will not be contended that the slight dilatation observable in the vessel was sufficient to call for a ligature—the idea is preposterous. We repeat, then, that we do not think that Mr. Wardrop exercised a due degree of caution, or did justice to his own high character, in deciding on the operation so hastily as in this case he appears to have done.

Another point, and that a very interesting one, remains to be considered, namely, the non-obliteration of the artery.

From an attentive consideration of all the circumstances, we are forced to conclude that the ligature was not applied to the artery. The blame we know has been laid upon the silk-worm gut, but this proved quite effectual in Mr. Lambert's case, which we shall detail presently, and in a case of Mr. Wickham's, which our readers will find in the Winchester Hospital Report of this Quarter. It may be said that the ligature slipped, but of this there is no evidence whatever. The Lancet, indeed, with great simplicity, conjectures "that the ligature gave way at the time of the retchings, about the twentieth day!" So that a thread may remain on an artery for nearly three weeks, *slip*, and leave no mark whatever behind it! When ligatures give way on the "twentieth day," it is commonly by a process called ulceration. We think, then, (for we have no alternative) that the carotid was never tied; but we are sure that no man, who knows any thing of the difficulties and uncertainties which attend operations on the arteries, and indeed almost all capital operations, will be very ready to decry Mr. Wardrop for committing, what none of us are exempt from, a *mistake*. Let him who is without fault throw the first stone.

If there be no error in the statement, that the superior thyroid and vertebral arteries were filled with a plug of "organised lymph," all we have to say is, that we can form to ourselves no satisfactory explanation of the circumstance. Whilst, however, we confess our own dulness, we are ready to acknowledge the acuteness of the Lancet. That able Journal settles the point forthwith. We cannot forego the pleasure of quoting the passage in question, a passage, we will venture to affirm, unequalled for the soundness and originality of its views of aneurism.

"It will be seen, that the ligature remained on *a sufficient length of time* to obliterate the *thyroid* and *vertebral* arteries, but *not long enough* to plug up the larger calibre of the carotid!" P. 21.

It has been imagined, we believe, by a few authors of no note, one John Hunter, and a Mr. Hodgson, and the opinion has, oddly enough, become general in the profession, that the effect of a ligature on an artery is to enlarge the collateral branches, in order (so the theory ran) to keep up the supply of blood to the limb. Thus, in the case of a ligature on the carotid, it was generally believed, that the superior thyroid, which comes off from it, and the inferior thyroid, which arises from the subclavian, would dilate, and become the principal channels of the new circulation. Indeed, not only was this the opinion of surgeons, but such was their infatuation, that they actually got up anatomical preparations to shew the fact. What a different view of the affair is taken by the Lancet. That Periodical informs us, that the first effect of tying the *carotid*, is *obliteration* of the *superior thyroid* and *vertebral*, (a branch of the subclavian!) the ligature not remaining on long enough to plug up the carotid itself!! Seriously, can the profession tolerate in a Medical Journal such matchless absurdity as this? Are the reputations of a Cooper, a Guthrie, a Brodie, or a Bell, to be for ever at the mercy of a Magazine which is utterly ignorant of so simple a matter as the effect of a ligature on an artery?

We shall now consider a third case of ligature beyond the tumour, performed on the carotid artery, by Mr. Lambert.

*Case.* A lady, æt. 49, of unhealthy appearance, presented a pulsatory tumour on the right side of the neck, immediately above the sternal end of the clavicle, partly covered by the mastoid muscle. It appeared to be the size of a walnut, and had all the characters of aneurism, but, on examining it accurately, it appeared to spring from the chest. Pressure on it occasioned much pain. Two years previously, the patient had experienced a severe mental shock, and, ever afterwards, on making any bodily exertion, she had tremblings and palpitations of the heart. These symptoms had increased so much, that, at the time of her application, she was incapable of pursuing her ordinary domestic employments. When these attacks came on, the respiration was most distressing, and on stooping she had a sense of suffocation, as if something pressed on the lower part of the windpipe. There was dryness in the throat, with occasional cough—sleep disturbed by frightful dreams—appetite bad—emaciation. The heart's action was so great, that it could be felt in any part of the chest—pulse at the wrist, as also in the carotids, vibratory. The beating of the right common carotid was very distinct, and it was this pulsation which first drew the patient's attention some months ago, the tumour not appearing till afterwards.* Mr. L. concluded that the tumour was an aneurism affecting the lower part of the right carotid artery, but whether confined to that vessel, or extending into the innominata, he could not tell. Sir Astley Cooper was consulted, and discountenanced the operation, remarking, that "*it was an aneurism by dilatation, which would not increase*," how truly will be seen in the sequel. Mr. Key thought that the innominata was affected, and that the operation was, therefore, inadmissible. Such, also, was the opinion of Mr. B. Cooper and Mr. Callaway. Mr. Wardrop was in favour of the operation, and Mr. Wakley recommended its immediate performance, which seems to have settled the question.

On the first of March, the operation was performed, in the presence of Mr. Wardrop, Mr. B. Cooper, and Mr. Callaway. The artery was laid bare above the tumour; it was unusually large, but not, to all appearance, diseased. One ligature of Fisherman's silk was thrown around the vessel, and the ends cut off close to the knot. The wound was then closed. The operation was performed without much difficulty; vomiting occurred a short time afterwards, but twenty drops of the vinum opii being administered in the evening, the irritability of the stomach went off. Immediately after the application of the ligature, the bulk of the tumour was evidently diminished, and its pulsation lessened. Next day, the vascular action was much more moderate than before the operation. The patient observed that "the beating of the heart was gone." On the third day, on removing the dressings, the tumour was greatly reduced in size, and only evident by a feeble pulsation. Her sleep was not disturbed, as formerly. On the tenth day, two or three drachms of blood escaped from the wound, the upper part of which had adhered, whilst the lower was suppurating freely. The application of linen, wet

* We apprehend that, in true aneurism, the reverse is mostly the case, the tumour appearing first without any distinct pulsation; indeed, the patient is frequently aware of none till it is pointed out to him.

with cold water, and strips of adhesive plaster, prevented any return of the hæmorrhage, and the report leaves the lady doing very well. This was on the 24th of March, and in the Lancet of the 19th of the succeeding May, the termination of the case is given. Her health continued, till the 17th of April, much improved, but, on that day, she complained of uneasiness and tingling about the wound, and in the centre of the cicatrix there was still a luxuriant granulation, little larger than the blunt end of a probe. Next day there was considerable hæmorrhage from the wound, but it was checked by the application of wet cloths. Bleeding again on the 19th, and at intervals until the 23d, from which time till the 30th it did not return. On the 1st of May, the hæmorrhage returned with great violence, and, at 11, A.M. the patient expired.

Twenty-four hours after death, the body was examined. Mr. Callaway was present, and Mr. Pilcher conducted the examination, and made a memorandum of the appearances. No tumour was visible in the neck externally. More than half the cicatrix had ulcerated, and a little below the external wounds there was ulceration, extending through the platysma myoides to the artery. On laying open the pericardium, there was seen "a thick layer of fibrine on the investing portion, (said to be common to rheumatic patients."*) The lungs and heart were healthy, the arch of the aorta "slightly" enlarged. The innominata externally was natural, on opening it, a few small patches of curdy matter were seen under its lining membrane. At the root of the right common carotid was a pyramidal tumour, about *half an inch* in breadth at the base, and extending two inches up the artery. Neither a probe nor water could be forced upwards from the innominata. On making a section of the tumour, its lower part was found accurately closed by a firm coagulum, which had formed the obstruction to the water. The coats of the artery round this coagulum were about four times their natural thickness, and lined by a thin layer of fibrine. Above the coagulum, they were six times their natural size, and besides the fibrine were lined with three layers of coagulable lymph. Where the ligature was applied, was an ulcerated opening on the anterior and tracheal surface of the carotid, a quarter of an inch in length, and less in breadth, covered with dark-coloured lymph, and communicating with the external wound. Opposite to this, on the posterior surface of the artery, was coagulum, on removing which, the division of the inner coats, produced by the ligature, was observed. Above the artery was not obliterated, but completely pervious, and studded with a few spots, like the innominata. Water thrown into the arch of the aorta, passed, by means of the superior thyroid, &c. into the *right* carotid, and out at the external opening.

Here, then, is another case of ligature beyond the tumour, and there can be no doubt that the ligature itself was effectually applied. It now remains

* This appearance is only common in such rheumatic patients as happen to become affected with pericarditis—a disease under which the present patient laboured, and under which she would most likely have fallen at no distant date. Indeed, the symptoms of greatly disordered function of the heart were unequivocal in this case from the beginning.

for us to consider whether or not there was an aneurism (a *true* aneurism) of the carotid. We have not the slightest hesitation in asserting our opinion that there was not, and that Sir Astley Cooper was perfectly right, when he stated it to be "an aneurism by dilatation." The history was more that of hypertrophy of the heart or large vessels, than of true aneurism, whilst the distressing dyspnœa, palpitations, and general ill-health, were out of all proportion to the size of the aneurismal tumour. The "rapid decrease of the tumour," on the application of the ligature, proves, what? that it was a sac filled with layers of coagulum? Certainly not; for, even in the common operation, where the *supply* of blood is, to a certain extent, arrested, no such "rapid decrease" takes place. Mr. Lambert, however, views the matter in a different light, for he advances this very circumstance as proof of aneurism! We doubt whether Mr. L. will get many to see the case through his glasses. But what we rely upon most of all is the plate published by Mr. Lambert himself, in No. 194 of the Lancet.

If that be not a representation of a *dilated* artery, we know not what is. The ligature, it is evident, has been applied just *above* the dilated portion, and, of course, the deposition of coagulable lymph, and formation of coagulum *in* that portion, have been the consequence. It may be said that absorption had been going on, and that the tumour was thereby much diminished in bulk. To this objection we reply, that there is no reason whatever to believe that diminution of size, to any extent, had taken place, for, at the time of the operation, the tumour was, as far as could be ascertained, about the size of a walnut, and, at the dissection, it was "half an inch in breadth, and two inches in length," a diminution no greater than what would certainly take place, though the artery were merely dilated. Our own opinion, then, is, that, in this case, there was no true aneurism of the carotid artery. But, even granting that there was aneurism, there were still cogent reasons against an operation. The patient was much out of health—there were symptoms of disorder, if not disease of the heart—and it was impossible for any one to say that the innominata, or even aorta were sound. On dissection, there *were* found pretty strong evidences of pericarditis, with dilatation of the aorta, and patches of curdy matter in the innominata.*

We have all along been warm advocates for Mr. Wardrop's operation, but we must confess that the perusal of these two cases, out of the three in which it has been performed, will hardly raise its character with surgeons, amongst whom there has always been a degree of prejudice against it. We almost fear it will be found that, where the aneurism is so low in the carotid as to prevent the application of a ligature between

---

* We cannot part from this case without expressing our dislike of the flippant, self-sufficient manner in which it is drawn up. The author sneers at Aston Key, and seems to think himself a very clever sort of gentleman. Perchance, he may learn for the future not to imagine himself wiser than Sir Astley Cooper, even though he should be backed by the high authority of Mr. Wakley.

it and the heart, there will be found disease of the innominata, aorta, or even heart, to boot, any one of which circumstances would, of course, turn the odds greatly against the operation.

P.S. Whilst this was in the press, we received the Medical and Physical Journal for June, in which there is a paper on this subject by Mr. Shaw. While we differ from him on one or two points, we are happy to find that, in the sentiments we have expressed, we are, for the most part, fully borne out by that gentleman. Mr. S. takes precisely the same view of Mr. Lambert's case with ourselves, and is of opinion that there was no aneurism. Every one who looks dispassionately and carefully to the history of the case and to the drawing, must, we are convinced, come to the same conclusion.

---

### 16. OBSTRUCTION OF ARTERIES.

[La Pitié.]

On several occasions we have taken notice of inflammation and obstruction of certain *venous* trunks, with the fatal consequences which often result therefrom. At present we have to advert to some instances of *arterial* obturation, brought forward by M. Bally, Physician to La Pitié.

*Case* 1. Diedan, aged 68 years, entered the hospital on the 3d of October, 1826, and died on the 18th of the same month. He had been affected with several attacks of cerebral congestion—he presented an extended "mucous wheeze," (râle muqueux) when examined by the stethoscope—the pulse was three to each interval of respiration, intermittent and irregular—some slight whizzing noise was heard attending each contraction of the left ventricle—impulsion moderate—general œdema. A few days after his entrance into the hospital, he complained of coldness in the left lower extremity, and no arterial pulsation could be felt in this limb. Within a few days of his death, the member turned livid, and was covered with gangrenous phlyctenæ, and desquamation of the cuticle.

*Dissection.* Immediately below the division of the aorta, the left iliac artery was obstructed by coagulated blood, the clot not appearing to be of ancient date, since it was still black. It was continued into the popliteal artery. The colour of the arterial coats resembled that of veins, but they were not diseased. The pericardium contained some sanguinolent fluid—heart greatly dilated in its ventricular chambers, and attenuated in its parietes, which tore with the greatest facility, and containing many fibrinous clots. The orifices and valves were in their natural condition. The aorta presented several osteo-cartilaginous elevations, which might, in some degree, account for the noise heard at each ventricular contraction—a phenomenon which M. Bally has several times found to depend on these aortic inequalities alone. The stomach was cancerous at its *cardiac* orifice, and ulcerated there, the disease spreading an inch into the œsophagus. The patient never had had any vomiting. The brain and other organs were sound.

*Case* 2. (Hôpital Cochin.) Margaret Tesson, of good constitution, aged 47 years, was ill for two days previously to her entering the hospital, 27th November, 1826. She complained of a paralytic attack of the right arm, which was painful, and incapable of motion, without much uneasiness. There was no indication of cerebral or spinal affection, and the complaint was considered as of a rheumatic nature, the patient being subject to rheumatism. On examining the radial artery, it was found that there was no pulse in that arm, except close up to the axilla. The circulation was natural in the other arm. The temperature of the right arm was rather lower than that of the other, and the sensibility much below par. The nails of the fingers, in this hand, were livid. At 7 o'clock the same evening, this patient dropped down and suddenly expired.

*Dissection.* The brain, spinal marrow, and the nerves of the right arm were perfectly healthy. The heart was of its natural size, the right auricle and ventricle full of black blood, as were the left chambers. The opening between the auricles still existed, admitting a free communication. It did not appear to be the effect of violence, but the original opening remaining unclosed. In the right axillary artery, there was found a very firm fibrinous clot, two inches in length, completely obstructing the flow of blood into that arm. The artery, just behind the obstruction, was somewhat dilated. The arterial parietes were sound. All the other organs of the body were in a healthy condition.—*Journ. Gen. de Medecine.*

---

### 17. AMPUTATION DURING THE PROGRESS OF MORTIFICATON.

This case happened lately at St. George's Hospital, and as it involves an interesting question in surgery we shall give a concise account of it.

Joseph Sparks, æt. 15, was admitted April 16th, 1827, under the care of Mr. Brodie, for a compound fracture of both bones of the left leg, occasioned by jumping over a ditch. The bones were broken at about their middle and the injury, to all appearance, not extensive. There was some pain at the head of the fibula. The lad was thin and sallow, and had suffered from rheumatic fever. The limb was put up in junks and union of the external wound attempted. On the 18th he complained of severe pain in the leg; this was attributed to the tightness of the bandages and they were loosened in consequence; the uneasiness however, continuing, on the next day, they were cut away, and some simple dressing applied. There was at this time a certain degree of anxiety in the countenance, and a slight sensation of numbness in the foot, but as there was nothing particular in other respects, little notice was taken of the circumstance. By the next morning, 20th, mortification had appeared in the leg commencing at the seat of the fracture, and extending thence downwards to the foot and upwards to the knee.

The features were sunken, the pulse hurried, and there was a wildness in the manner, not amounting, however, to incoherence or delirium. The gangrene continuing to spread, it was determined to give the boy the only chance which remained, viz. amputation. Before proceeding to the operation it was remarked that there was an emphysematous crepitation in the thigh particularly at the back part—that the cutaneous veins appeared loaded, and that the glands in the groin were enlarged. The tongue was dry in the centre, but the pulse though quick was steady. At 4 P. M. the limb was removed a little below the trochanters, as high as could conveniently be done, but on cutting through the cellular membrane, it was found to be of a brownish yellow colour, and infiltrated with serum, in fact disposed to take on the gangrenous state. The lips of the stump were brought together by sticking plaster, &c. and the patient placed in bed. He bore the operation very well, and on its completion the anxiety of the countenance subsided remarkably.

On examining the amputated leg, all the parts were found more or less disorganized, especially the muscles and cellular tissue. The fracture was not very extensive, but the fibula was dead and completely separated from the periosteum in its whole length from the epiphysis to the malleolus. Indeed, on slitting open the periosteum, the bony shaft fell out! The same, but not to quite so great an extent, was the case with the tibia. The epiphyses themselves of both bones were unaffected. In the cavity of the knee-joint there was, we believe, some suppuration. The neurilema of the sciatic nerve was studded with little patches of ecchymosis. Nothing particular was observed about the blood-vessels.

21st. He was rather delirious yesterday afternoon, but slept well in the night; he did not complain of much pain and was perfectly sensible. On waking at 8 o'clock this morning, the jaw was seen to be locked in some degree; the stump began to start now, and the countenance shewed considerable anxiety, as the morning advanced, the tetanus became fully marked—opisthotonos appeared—the pulse became wiry and small—and at 10 P. M., in spite of all means, the exhibition of the prussic acid, &c. the poor boy died.

No dissection was permitted, or at least, wished by the friends. On looking at the stump, there was no appearance of mortification, nor was there any attempt at adhesion.

*Remarks.* The pain in the first instance at the head of the fibula, seems to shew that the epiphysis was torn from the shaft by the shock of the accident; such, too, in all probability, was the case with the periosteum to a certain extent; but the complete separation which took place of this membrane from the bone must have been principally owing to inflammation. It is curious that in the case of traumatic gangrene related by Mr. Guthrie in his work on Gun-Shot Wounds, where amputation was performed by Mr. Campbell, the periosteum, as here, was found separated from the tibia and fibula. It is well known that in gangrene, as in erysipelas, the cellular tissue, from its low degree of vitality, suffers most. This fact was well illustrated in the present case, for in the thigh, where all the other textures were sound, or nearly so, the cellular membrane was disorganized, and almost gangrenous. It is difficult in this instance to account satisfactorily for the occurrence of the mortification. The injury to the parts was by no means particularly severe, nor were any of the great vessels ruptured. The *shock*, however, it must be remembered, was considerable, and the poor boy's constitution was enfeebled. We now come to the question of amputation. We think it was justifiable, nay more, perfectly judicious. At the same time we question whether the result proves much in any way. It certainly does not prove *against* the operation, for the patient died of tetanus, and that was a *casualty* that no one could or can count on. On the other hand, it does not prove much *for* the operation, for if the mortification were to come upon the stump, it is more than probable that it would not appear within the first 36 or even 48 hours. We repeat then that this case bears little upon the propriety or impropriety of amputating during the spreading of traumatic gangrene—a question deeply interesting in point of principle, and important in practice—a question which is yet to be settled by the experiments or experience of the profession.

---

### 18. ON HYPERTROPHY OF THE MUSCULAR COAT OF THE STOMACH. BY DR. BOUILLAUD.

[Hôpital Cochin.]

Every pathologist is familiar with two very different diseases of the heart—dilatation of the cavities with *thickening*, and dilatation with *atrophy* of the parietes. The very same pathological conditions obtain in the stomach—and probably from the very same causes. What are these causes? In respect to the heart, we know that contractions of the origin of the pulmonary artery and of the aorta are usually accompanied by, and probably cause enlargements of the corresponding ventricles, with or without thickening of the parietes. But we know, also, that enlargements of the heart take place without any obstruction at the roots of the large arteries, and consequently without any demand being made on the muscular structure of the organ for any increase of action. In these last cases, we generally conclude that a chronic inflammation has settled on the heart, and produced an overplus of nutrition in the organ. But how shall we account for atrophy of the muscular structure, with dilatation of the cavities? Can the same cause produce diametrically opposite effects? Strange and illogical as this last position may appear, we are of opinion that, in the animal economy at least, it is far from untrue.

But to return to the stomach. We very often find that, when scirrhus has invaded the pyloric orifice, the stomach becomes enlarged in its dimensions—sometimes with thickening of the muscular coat—sometimes with atrophy of the parietes of the organ. Modern pathologists have attributed this dilatation and hypertrophy to the obstruction of the pylorus, and consequently to the exertion which the stomach makes to unload itself, either by the pyloric orifice, or by vomiting. The theory is specious, and is maintained by Louis, Andral, and others; but it is not without its difficulties requiring further investigation. M. Bouillaud has recently published two cases bearing upon this question, which we shall briefly notice.

*Case* 1. A perfumer, aged 40 years, was received into the Cochin Hospital, on the 28th June. Two years previously he had been ill of a complaint attended with vomiting and pain in the epigastrium. When examined in the hospital, he presented the following symptoms, viz: pain throughout the whole epigastric region, across which was felt a kind of elastic body, suspected to be the great arch of the stomach thickened—the pain in this part had been gradually increasing for the last six months—abdomen voluminous and fluctuating—tongue red and moist—thirst ardent—no material loss of appetite—nausea and sense of burning heat in the stomach after eating, followed by vomiting of almost all the ingesta, at various intervals after each repast. During the preceding two months the patient had not passed more than three or four stools, and those obtained by lavements—countenance sunk and expressive of suffering—skin dry, pulse quick and small—marasmus much advanced. *Diagnosis.* Cancer of the pylorus. Lemonade—lavements. During the month of July, the patient had frequent motions of a black colour. The vomitings continued, and the matters ejected were often like coffee-grounds. He complained of lancinating pains in the epigastrium. Ascites became unequivocal, accompanied by œdema of the lower extremities. He lingered out till the 9th August, when he expired.

*Dissection.* The peritoneal cavity contained a considerable quantity of yellowish serum, and the peritoneum itself presented numerous tubercles on its internal surface—stomach distended by an enormous mass of vegetable aliment, mixed with a vinous looking fluid exhaling a very acid odour—pyloric orifice so contracted that the finger could not be pushed through it, the contraction occasioned by thickening of the parietes and numerous fungous excrescences. The very small passage that remained was blocked up by stones of fruit and other indigestible substances that had been swallowed. The three coats of the stomach, in the neighbourhood of the pylorus, were confounded into one lardaceous mass, as is commonly seen in cases of scirrhous pylorus. Throughout the rest of the organ the mucous membrane presented numerous traces of inflammation, and even erosion. The muscular coat was developed to a most extraordinary extent. The muscular fibres were precisely like those of a voluntary muscle, red and distinct. There was nothing particular in the other abdominal viscera. In the thorax, the lungs presented many tubercles, and the pleura was thickened and diseased. The heart was not more than half its natural size; but its chambers were well proportioned.

*Case* 2. Ann Bruneau, aged 55 years, was received into the hospital on the 19th November, having been ill for 13 months, occasioned, as was supposed, from troubles of mind. Loss of appetite, pain in the stomach, vomitings, and occasional diarrhœa were the principal symptoms which the patient detailed, as heretofore obtaining. She was now much emaciated, countenance and skin generally of a yellow tint—tongue red, clean, and moist—pain in the epigastrium—thirst—vomiting of almost every thing eaten—diarrhœa—colicky pains—tenesmus—skin cold—frequent shiverings, or at least chills—pulse quick, small, and soft—tendency to faintness. *Diagnosis.* Cancer of the pylorus. She lingered till the 19th December, and then died, 40 days after her entrance into the hospital.

*Dissection.* The peritoneal cavity contained a considerable quantity of greenish fluid, with flocculent matters floating in it. The intestines were agglutinated into a roundish mass by means of false membranes. The stomach was greatly distended by alimentary matters, and its coats were so lacerable, that the fingers readily went through them. The pyloric orifice was surrounded by a scirrhous induration, in which all the tunics were amalgamated into one homogeneous lardaceous substance, the internal surface of which was in a state of ulceration. The muscular coat of the organ was in a state of development or hypertrophy, as in the preceding case. The pyloric passage, however, was sufficiently open to admit a finger to pass freely into the duodenum. Both in this and the preceding case, the size of the stomach was greatly beyond the natural volume of that viscus, and seemed in proportion to the hypertrophy of the muscular tunic.

Two cases have been published by M. Louis, in which the stomach was greatly enlarged in capacity, and the muscular coat in a state of hypertrophy. As we stated before, we have no other means of accounting for this pathological condition, but by the efforts which the stomach makes to eject its contents, whether upwards or downwards—or by the circumstance of chronic irritation and inflammation in the organ, by which an afflux of blood is drawn to the part, and its structure thus morbidly or inordinately nourished. The very same exertions to overcome resistance or evacuate the contents of the organ—and even the same chronic inflammation, might, in particular constitutions, give rise to softening, dilatation, and thinning of the coats of the stomach, as we see in the heart. Some people will have active, and others passive dilatation of the central organ of the circulation, the causes and attendant circumstances being apparently the same in both cases.

---

### 19. GASTROTOMY FOR STRANGULATED INTESTINE.

[St. George's Hospital.]

A very interesting case occured lately in the practice of Mr. Brodie, at St. George's Hospital.

A surgeon was summoned on the 22nd of April, at six o'clock, a. m. to the patient, a married woman, æt. 35, for what was called "miscarriage." He found her in great pain, and from the anus there protruded a vast heap of *small intestine*, to which much violence had been done by the attendants in their attempts to return it, or bring it away! It appeared that the woman had been subject to prolapsus ani, that she had been drunk the night before, and that she had had a good deal of vomiting. The gentleman, Mr. Mullins, tried to reduce the protrusion, but though it could be pushed up within the rectum, it soon descended again. Mr. Brodie was now called in, and he sent the patient to the hospital. On examination there, he found between five and six feet of small intestine protruding through the anus. The gut was highly injected and coated with coagulable lymph, and in it was a perforation apparently caused by the handling to which it had been subjected. This was secured by a ligature, and some ineffectual efforts made to reduce the hernia. On passing the finger up the rectum a lacerated opening was found on its anterior part, at the distance of about two or three inches from the anus. Through this the mass of intestine had protruded, and through this it was now attempted to return it, but in vain.

Mr. Brodie considering that, if nothing was done to relieve the strangulation, death must inevitably result, determined to give the patient the only chance left, though that chance was, of necessity, but a desperate one. He accordingly made an opening through the linea alba into the abdomen—the intestine was then pulled up through the slit in the rectum and replaced in the abdomen—and the lips of the external wound were brought together by sutures, with a compress and bandage over all. This was at seven p. m. The symptoms of inflammation however did not abate, at least to any extent, and by the next morning the pulse was quick and small—the pain in the belly horrible—the countenance sunk and inexpressibly anxious—the extremities below their natural temperature, and at six p. m. 23 hours after the operation the woman died.

*Dissection.* On laying open the abdomen, the ileum, to the extent of five or six feet or more, was seen to be injected and in parts highly inflamed, its convolutions being agglutinated by lymph. The inflammatory appearance, notwithstanding, was by no means so marked, as before the operation. In the cavity of the pelvis, fæcal matter was found extravasated, and on the anterior surface of the rectum was seen the opening through which the hernia had taken place. This opening had all the characters of a laceration, though its lower edge had more the appearance of being rounded off by ulceration, which was probably the mere effect of the pressure and efforts at reduction. None of the other cavities were examined.

There is no very satisfactory way of accounting for the laceration in the rectum; what followed of course is intelligible enough. It was imagined by some, that there had been previously an ulcer, at this part of the intestine, which had cicatrized, and that during the vomiting this cicatrix had given way; but this is pure conjecture, and not at all warranted by the appearances on dissection. Whether the part had been weakened by the previous difficult labours, (for the woman had had several) or by the prolapsus, is more than any one can with certainty say. The treatment was bold and decided, and does no discredit to the reputation of Mr. Brodie.

The Lancet, we perceive, with its usual candour, whines against this excellent surgeon for having performed the Cæsarian Section for Prolapsus Ani!!

